# Paternal genome elimination promotes altruism in viscous populations

**DOI:** 10.1111/evo.14585

**Published:** 2022-08-07

**Authors:** Thomas J. Hitchcock, Andy Gardner

**Affiliations:** ^1^ School of Biology University of St Andrews St Andrews KY16 9TH United Kingdom

**Keywords:** Dispersal, haplodiploidy, inbreeding, intragenomic conflict, paternal genome elimination, social behavior

## Abstract

Population stieveness has lang been thocht tae forder the evolution o altruism. Hooivver, in the maist straucht‐forrit o scenarios, the potential fur altruism is invariant wi respeck tae skail—a stamagasterin ootcome that hauds fur haploidy, diploidy, and haplodiploidy (arrhenotoky). Here we pit forrit a kin‐walin model fur tae airt‐oot hoo population stieveness affects the potential fur altruism in species wi male paternal genome drap‐oot (PGD), takkin tent o altruism enactit by baith females and males, forby baith young‐anes and aulder‐anes. We find that: 1) PGD forders altruistic ongauns relative tae the ither inheritance seestems, forby tae a degree that depends on the extent o paternal genome kythin. 2) Unner PGD, skail maks mair muckle the potential fur altruism amang young‐anes and gars it less likely amang aulder‐anes. 3) The genetics o PGD can lead tae kenspeckle differences in sex‐specific potentials fur altruism, even wioot onie sex‐differences in ecology.

“over a range of different species we would expect to find giving traits commonest and most highly developed in the species with the most viscous populations.”

– Hamilton (1964a)

“the point is that, to be effective, altruism must put offspring into competition with non‐altruists, not bunch them in a wasteful competition with their own kind.”

– Hamilton (1971)

Population viscosity has long been suggested to promote the evolution of altruistic behavior, because when individuals remain close to their place of birth during the course of their lives, they will often be closely related to their neighbors, such that even indiscriminate altruism will tend to primarily benefit their genetic relatives (Hamilton 1964a,b). However, alongside increased relatedness, population viscosity also increases the extent to which individuals compete with those same relatives for resources, that is, kin competition (Hamilton 1971, 1975; Alexander 1974; Frank 1998). Under the simplest of models—including the infinite, inelastic island model of population structure—these two effects of increased relatedness and increased kin competition exactly cancel, such that the rate of dispersal has no net impact on the level of altruism that is evolutionarily favored (Taylor 1992a,b; Wilson et al. 1992; Queller 1994; West et al. 2002). This finding has sparked a body of theoretical research into understanding when and why this cancellation effect may break down, examples of which include overlapping generations (Taylor and Irwin 2000; Irwin and Taylor 2001), budding dispersal (Gardner and West 2006), sex‐biased dispersal (Johnstone and Cant 2008; Gardner 2010), and density‐dependent dispersal (Kanwal and Gardner 2022), among others (see Cooper et al. 2018 for an overview).

The primary focus of this theoretical work has been on ecological factors, and relatively little work has been done to investigate whether alternative genetic systems may cause this cancellation result to break down. One reason might be that Taylor's (1992a) analysis, which launched this avenue of inquiry, already obtained results for haploidy, diploidy, and haplodiploidy (more specifically arrhenotoky), and found that the cancellation holds under all three genetic systems (Taylor 1992a). Although this might suggest that the cancellation result holds robustly in the face of variation in genetic system, more recent results hint that this need not be the case. Specifically, Yeh and Gardner's (2012) general‐ploidy version of Taylor's (1992a) original model reveals that the cancellation breaks down in unusual scenarios whereby one sex contributes genes to the other sex but not vice versa. Similarly, a recent model of the evolution of male harm investigated cases of imperfectly uniparental transmission of cytoplasmic genes, finding that this, too, results in social behavior that is not invariant with respect to the rate of dispersal (Hitchcock and Gardner 2021). However, the extent to which different inheritance systems may decouple viscosity's effects upon relatedness and kin competition remains obscure.

An understudied genetic system that may be of particular interest is that of male paternal genome elimination (PGE; Haig 2002; Burt and Trivers 2006; Gardner and Ross 2014; de la Filia et al. 2015; Hodson et al. 2017; Jaron et al. 2022). Under this system—which is found in groups of flies, springtails, mites, coccids, and beetles—males receive, but do not transmit, a paternal genome. This paternal genome, although not transmitted, may nonetheless influence the phenotype of the male, with the extent of this influence determined by the developmental timing of the paternal genome's elimination and the extent of the paternal genome's expression, factors that vary between tissues and species (de la Filia et al. 2015, 2018). Thus, although the transmission genetics of PGE are equivalent to “conventional” haplodiploidy (i.e., arrhenotoky), the somatic genetics differs, with both males and females being diploid. Recent years have seen increased interest in PGE systems, not only because they include economically important pests (e.g., the coffee borer beetle), but also because, with the advent of new genomic tools, their remarkable genetics enables potentially exceptional tests of evolutionary theory (Featherston et al. 2013; de la Filia et al. 2015; Klein et al. 2021; Hitchcock et al. 2022).

Here, we construct a kin selection model to investigate how population viscosity alters the potential for altruism in haploid, diploid, haplodiploid (arrhenotokous), and male PGE species. We consider altruism enacted by both males and females, and at both juvenile (predispersal) and adult (postdispersal) stages, allowing for various sex biases in demography. We find that (1) PGE promotes altruistic behaviors relative to the other inheritance systems, with the extent of this shaped by the degree of paternal genome expression; (2) unlike diploidy and arrhenotoky, dispersal does alter the potential for altruism in PGE species, with the direction of this effect dependent on the point in the life cycle that the altruism is expressed; and (3) PGE's asymmetric genetics can lead to striking differences in sex‐specific potentials for altruism, even without any further sex‐specific ecology being assumed.

## Methodology

We consider an infinite population subdivided into patches, whereby on each patch there reside a large number of juveniles born to *n* females and *n* males. These juveniles invest in a social behavior that modulates their survival to adulthood *S*, with a focal individual's survival being determined both by their own investment *x*_j_ and also by the investment of their social partners *y*_j_: specifically, we have ∂($S/\bar{S}$)⁄(*∂x*_j_) *= − c*_j_ for self and *∂*($S/\bar{S}$)⁄*(∂y*_j_) = *b*_j_ for social partners, where $\bar{S}$ is the mean survival of juveniles in the population. Individuals then disperse from their patch with probability *d*. Following dispersal, individuals compete for representation within the *n* breeding adults of each sex on each patch, with all unsuccessful individuals dying. Adults then engage in further social interactions that modulate their fecundity *F*, with a focal individual's fecundity modulated both by their investment *x*_a_, the investment of their same‐sex social partners *y*_a_, and of their opposite‐sex social partners *y*'_a_: specficially, we have ∂($F/\bar{F}$)⁄∂*x*_a_ = − *c*_a_, ∂($F/\bar{F}$)⁄∂*y*_a_ = *b*_a_, and ∂($F/\bar{F}$)⁄∂*y*'_a_ = *b*_a_ *−c*_a_, where $\bar{F}$ is the mean fecundity of adults in the population. After new offspring are born, the adults on the patch then die and the life cycle begins once more. This life cycle thus encompasses the model of Gardner (2010), which investigated the social behavior of juveniles, and the model of Johnstone and Cant (2008), which investigated the social behavior of adults, although without any sex differences in ecology. Further details on this life cycle and its associatced fitness functions, plus extensions to sexual asymmetries in both dispersal and the number of breeders, are given in Supporting Information S1–S3.

We determine the conditions under which natural selection favors an increase in the level of these two social traits using the kin‐selection methodology of Taylor and Frank (Taylor 1996; Taylor and Frank 1996; Frank 1998; Taylor et al. 2007). This approach analyzes how the relative fitness of a focal individual is altered by both small changes in their own trait value and by correlated changes in the trait values of their social partners, with the extent of phenotypic correlation being determined by their relatedness to those social partners (Supporting Information S4). These changes in relative fitness are then weighted by the reproductive value of the focal individual's class (Supporting Information S5). These methods assume that selection is weak and that there is vanishingly little genetic variation, in order that the powerful tools of differential calculus be brought to bear on the problem. For this analysis, we treat juvenile and adult social behaviors as independently evolving traits that may show sex‐limited expression.

As we investigate altruistic behavior, we restrict our attention to scenarios in which juvenile social behavior incurs a positive survival cost for self (i.e., *c*_j_ > 0) and provides a positive survival benefit for social partners (i.e., *b*_j_ > 0), and in which adult social behaviour incurs a positive fecundity cost to oneself and to ones mating partners (i.e., *c*_j_ > 0) and provides a positive fecundity benefit shared across the individuals in the patch (i.e., *b*_a_ > 0), although other combinations of fitness effects are possible. We can then use these marginal fitness effects (in conjunction with the appropriate relatedness and reproductive‐value coefficients) to calculate our conditions for increase (Supporting Information S6). We then rearrange these conditions into the form c_t_/b_t_ *<A*_t_, where *A*_t_ is the potential for altruism at time t in the life cycle (t∈{j,a}) (cf. Gardner 2010). With higher levels of *A*, it is less stringent for helping behaviours to increase, and more stringent for harming behaviours to increase. Further methodological details can be seen in Supporting Information S1–S6.

## PGE and the Potential for Altruism

**Figure 1:**
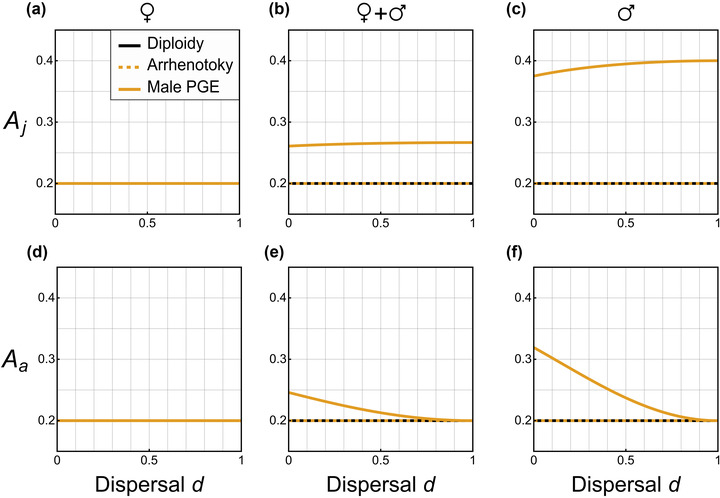
Dispersal modulates the potential for altruism (*A*) under paternal genome elimination (PGE), but not under diploidy or arrhenotoky, with the direction and magnitude of effect depending on when during the life cycle the behavior is expressed (a–c, juveniles *A*_j_; d–f, adults *A*_a_), and the sex of the actor expressing the behavior (a, d, exclusively females; b, e, both sexes; c, f, exclusively males). Across all panels *n* = 5. For the case of male PGE, we assume that there is equal expression from the maternal‐origin and paternal‐origin gene copies in males (i.e., τ = 1/2). Explicit expressions for all these cases and extensions to sex‐biased dispersal and patch size can be found in Supporting Information S6.

We begin by considering altruism enacted solely by females, that is, where the trait is exclusively expressed by females, although both males and females may be recipients of the behavior. For both juvenile and adult females, and for haploidy, diploidy, haplodiploidy (arrhenotoky), and PGE, we find that the potential for altruism is given by *A*_t_=1/*n*, where *n* is the number of male and female breeders on the patch, that is, the size of the demographic “bottleneck” that generates nonzero relatedness. That is, we recover the cancellation result as it pertains to female‐only altruism under haploidy, diploidy, and haplodiploidy (Taylor 1992a; Johnstone and Cant 2008; Gardner 2010; Johnstone et al. 2012), and show that it also extends to female‐only altruism under male PGE (Fig. 1a,d).

Next, we consider altruism enacted solely by males (Fig. 1c,f). For both juvenile and adult males, and for haploidy, diploidy, and haplodiploidy (arrhenotoky), we find that the potential for altruism is given by *A*_t_=*1*/*n*. That is, we recover the cancellation result as it pertains to male‐only altruism under these three genetic systems (Johnstone and Cant 2008). In contrast, under male PGE, we find that the potential for altruism amongst juveniles is

(1) $$A_{j}=\frac{2\left(4-{\left(1-d\right)}^{2}\right)}{4n-{\left(1-d\right)}^{2}\left(n-1\right)},$$

and among adults it is

(2) $$A_{a}=\frac{4n+{\left(1-d\right)}^{2}\left(3n+1\right)-{\left(1-d\right)}^{4}\left(n+1\right)}{4n^{2}-{\left(1-d\right)}^{2}\left(n-5\right)n-{\left(1-d\right)}^{4}\left(n+1\right)}.$$

Inspecting these equations, we make several observations. First, the potential for altruism is higher under male PGE than the other investigated inheritance systems. Second, the potential for altruism is higher for males than for females. Third, unlike in the other cases, the potential for altruism depends upon the rate of dispersal. Fourth, the effect of dispersal is qualitatively different for juveniles and adults: among juveniles, increased dispersal is associated with an increase in the potential for altruism, whereas among adults increased dispersal is associated with a decrease in the potential for altruism. These patterns can be seen in Figure 1. In the case where altruistic behavior does not show sex‐limited expression (Fig. 1d,e), then the altruism‐promoting effect of PGE in relation to males leads to both males and females exhibiting a potential for altruism that is both higher than that predicted for haploid, diploid, and haplodiploid (arrhenotokous) genetic systems and also dependent upon the rate of dispersal (Fig. 1; Supporting Information S6).

**Figure 2:**
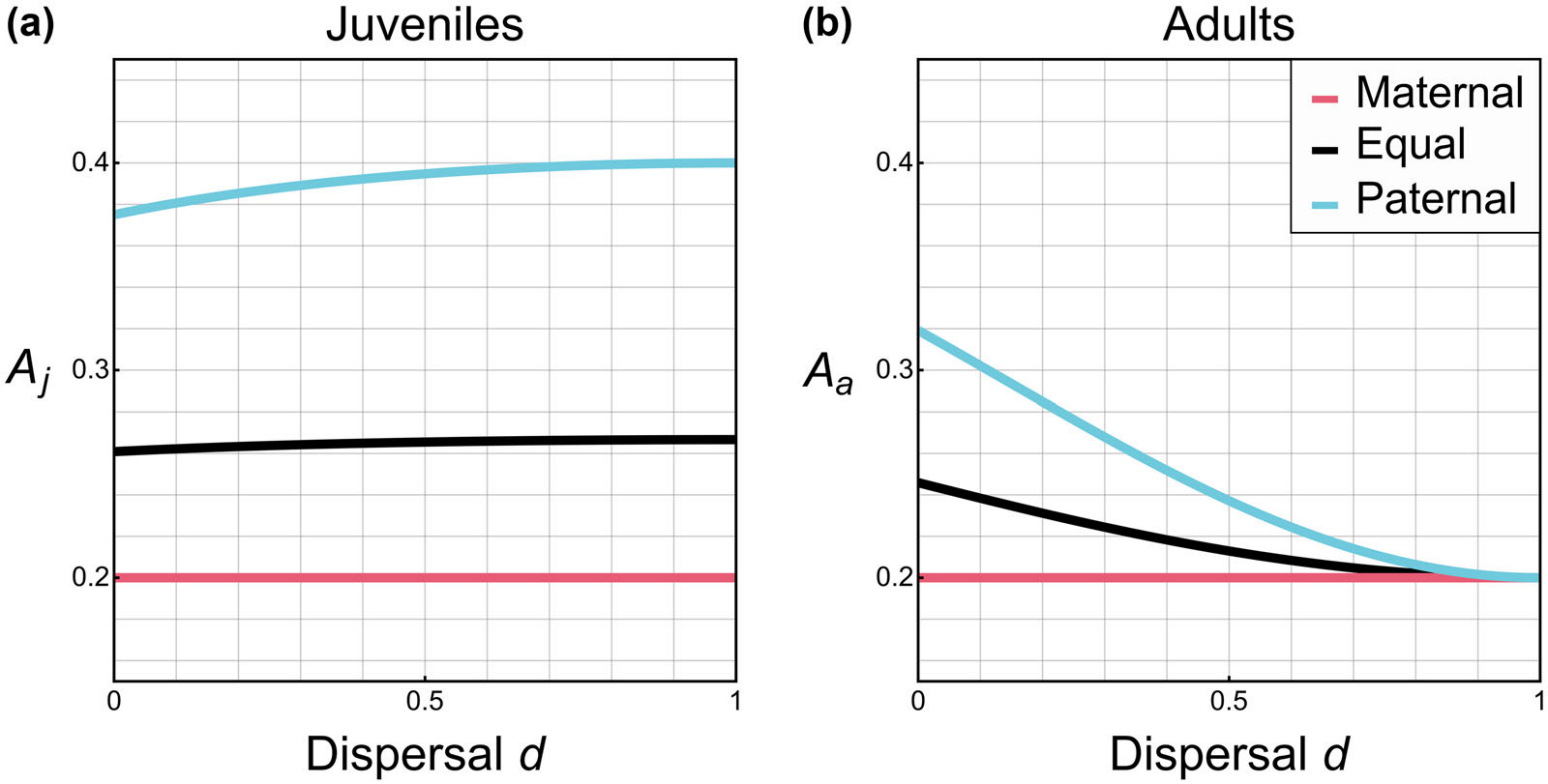
The extent of expression from the paternal‐origin genome modulates the potential for altruism (*A*) in males under PGE both at (a) juvenile (predispersal) *A*_j_ and (b) adult (postdispersal) *A*_a_ stages. With lowest altruism when there is exclusively maternal‐origin expression in males (τ = 0), higher potential for altruism with equal expression from those two gene copies (τ = 1/2), and the highest potential for altruism when there is exclusively paternal‐origin expression in males (τ = 1). In both panels *n* = 5. Explicit expressions for all these cases can be seen in Supporting Information S6.

These differences between PGE and arrhenotoky are, ultimately, due to the expression of the male paternal‐origin genome. As this genome is not transmitted by its carrier, it has no direct fitness interests in the reproduction of that carrier, and thus is predisposed to altruism. We can show this by altering the influence that the paternal‐origin genome has upon the male phenotype (Fig. 2; Supporting Information S4 and S6). This also allows us to explore some of the natural variation seen in the extent of male paternal genome expression (e.g., de la Filia et al. 2015). When the phenotype is exclusively controlled by maternal‐origin genes, that is, solely the maternal‐origin gene copy is expressed in males, the results coincide exactly with those for arrhenotoky, yielding A_t_=1/*n* for both juveniles and adults. In contrast, when the phenotype is under the sole control of the paternal‐origin genes, that is, solely the paternal‐origin gene copy is expressed in males, then the potential for altruism is higher still, with the same qualitative pattern as reported above (Fig. 2). Thus, we can also see that, due to their different potentials for altruism, there is scope for strong intragenomic conflict between the maternal‐origin and paternal‐origin genomes in males (Burt and Trivers 2006; Gardner and Úbeda 2017). Full analytical expressions can be seen in Supporting Information S6, and the additional effects of sex‐biased demography can be seen in Figures S2–S9.

## Discussion

Here, we have shown that the unusual genetics of PGE, working in combination with population viscosity, is expected to drive distinct patterns of social behavior as compared to other genetic systems that have been investigated previously. This includes generally higher levels of altruistic behavior, with the extent of this dependent on the timing of the social behavior, sex of the actor, degree of paternal genome expression, and—notably—the rate of dispersal. These effects owe to the relative disincentive faced by a male's paternal‐origin genome with respect to the pursuit of his personal reproductive success, on account of this portion of his genome not being transmitted to his offspring, and which therefore makes him more inclined to altruistic behavior. These results indicate that various PGE groups may prove to be exceptional study systems with which to investigate the evolution of social behaviors, lending themselves to clear‐cut within‐ and between‐population comparative predictions concerning these factors that do not apply in more standard genetic settings.

Previously, much of the work unpicking the classic result that the evolutionarily favored level of altruism is invariant with respect to the rate of dispersal has been focused on ecology. This, as suggested above, may stem from Taylor's (1992a) thoroughness in covering the most common genetic systems—haploidy, diploidy, and haplodiploidy (arrhenotoky)—and showing that the same result obtains in all cases. However, recent results demonstrate that there are genetic systems wherein this invariance does not hold (Yeh and Gardner 2012; Hitchcock and Gardner 2021), with our results providing yet another example. Some of these systems, such as those featuring the zero‐reproductive‐value “zombies” investigated by Yeh and Gardner (2012), are likely rare in nature, with the closest approximations of this being the hermaphroditism of Icerya (Gardner and Ross 2011) and the androgenesis of corbicula clams, Saharan cypress, and *Bacillus* stick insects (Schwander and Oldroyd 2016). PGE, by contrast, is more common, having arisen independently in at least seven clades of arthropods, and thought to be in many thousands of species. Given the findings of the present analysis, it is worth re‐examining some other unusual genetic systems that—even if rare—may provide other interesting exceptions to the invariance result. For example, species that exhibit somatic chimerism—such as Callitrichid monkeys (Haig 1999; Ross et al. 2007; Patten 2021), brown seaweeds (González and Santelices 2017), hydrozoans (Chang et al. 2018), and scleractinian corals (Puill‐Stephan et al. 2009; Schweinsberg et al. 2015; Guerrini et al. 2021)—share some conceptual similarities to PGE, with individuals containing genes that may not be transmitted further, and thus may also be worth investigating, both theoretically and empirically, in the light of this work. Moreover, unusual systems such as PGE provide interesting test cases with which to enrich our understanding of how relatedness, reproductive value, and kin competition intersect to shape the evolution of various social behaviors. This may prove useful for when we move beyond the comforts of classic population genetics to try and understand the consequences of stranger, nongenetic inheritance systems (Bonduriansky and Day 2018).

Population viscosity is also particularly relevant for PGE species that—like other haplodiploid groups—often experience ecologies involving significant population subdivision, limited dispersal, and high levels of inbreeding (Hamilton 1967; Burt and Trivers 2006; Gardner and Ross 2014; Hitchcock et al. 2022). Although here we have focused on a generic life cycle to illustrate the difference in the potential for altruism between PGE and other inheritance systems, future modeling should incorporate more of the idiosyncratic life cycle features found in these groups, as well as the variation among them. Such details might include the timing of mating during the life cycle, the extent of generational overlap, and monogenic reproduction. These details will not only enrich the theory but will also enable more ecologically relevant models to be tailored to these particular groups.

The present analysis suggests that we may expect PGE species to display distinct patterns of social behavior. However, this is currently challenging to test as data on the social ecology of some of these groups remain relatively sparse. This is in part be due to technical issues, as many of these species are small, and often live in harder‐to‐view locations such as within soil or under bark. Nonetheless, there are some interesting instances of quite striking social behaviors. For example, since the 19th century, strange mass movements of the larvae of sciarid flies (primarily *Sciara militaris*) referred to as “armyworms” or “snakeworms” have been observed in Europe, North America, South America, and Asia (Sutou et al. 2011). Additionally, some groups have unusual mating behaviors, such as those described in globular springtails (*Deuterosminthurus bicinctus*) whereby males and females engage in a “push‐and‐pull” courtship ritual, followed by sperm transfer, and then competition between mates for spermatophore remains (Kozlowski and Aoxiang 2006). Alongside further study of particular social behaviors, groups such as the scale insects may be particularly amenable for comparative tests as to how mode of inheritance shapes social behavior, with this group spanning an extraordinary array of genetic systems, from diploidy and arrhenotoky, to male PGE and even hermaphroditism (Nur 1980; Ross et al. 2010; Mongue et al. 2021).

We have also shown that the asymmetric genetics of PGE generates strong sex differences in the potential for altruism, which may be associated with strong sex differences in social behavior and concomitant sex‐specific morphologies. One interesting behavioral pattern that qualitatively aligns with our results is seen in the armored scales whereby male crawlers feed on exposed and dangerous leaves, whereas females feed in the more‐protected crevices in the bark (Gill 1997 ; Normark 2004 ). This could be viewed as an altruistic behavior by juvenile males to alleviate kin competition, although this has also been suggested to be driven by matrilineally inherited endosymbionts (Normark 2004; Ross et al. 2010). In *Cystococcus* coccids (Eriococcidae), female crawlers are carried to new feeding sites by their older, alate, brothers, with a single male carrying as many as 13 female crawlers (Gullan and Cockburn 1986). This intersexual phoresy has also been suggested to occur in three other groups of gall‐inhabiting coccoids: *Mangalorea*, *Gallacoccus*, and *Echinogalla* (Takagi 2001). The males of these gall‐forming coccids also display some further intriguing features, such as robust legs and elongate, sharp claws, and thus the male nymphs have been suggested to play a defensive role (Takagi 2007). Second‐instar males have also been suggested to perform a similar defensive role in the genus *Rutherfordia* (Takagi 2021).

More generally, these results may be linked to the extreme sexual dimorphism observed in some of these groups (Gray 1954; Damon 2000; Palacios‐Vargas and Castaño‐Meneses 2009). Such sexual dimorphism may, in turn, also modulate conditions for social behaviors to evolve (e.g., sex‐biased dispersal; Johnstone and Cant 2008; Gardner 2010; Johnstone et al. 2012; Supporting Information S6), and thus further modeling is needed to understand how these factors may coevolve with one another. For example, if in PGE species males evolve to be less competitive with their siblings than are females, or provide a defensive role for the nest, then this may shape the sex‐allocation decisions of parents. This is conceptually similar to models that have investigated coevolution of sex‐specific offspring helping and sex allocation (Gardner and Ross 2013; Davies et al. 2016). In addition, if such sex‐specific strategies are favored, but sex‐limited expression is not possible, then this may generate sexual antagonism, which is known to manifest differently in PGE species (Klein et al. 2021; Hitchcock et al. 2022) and may also be altered by sex‐biased demographic processes (Flintham et al. 2021; Hitchcock et al. 2022), further altering evolutionary trajectories.

We have also considered how, within males, maternal‐origin and paternal‐origin genes may have very different potentials for altruism. This might be expected to lead to intense intragenomic conflicts of interest over a wide class of social traits, in addition to the conflicts that exist over transmission (Herrick and Seger 1999). Previously, Ross et al. (2011) investigated one such conflict, modeling how a paternal‐origin‐expressed male suicide trait may invade a population, generating a selection pressure for the silencing of the paternal genome from the maternal‐origin genome. They suggest that this may be one explanation for the common pattern of paternal‐genome heterochromatization seen in PGE groups. Given that we might expect strong intragenomic conflict between these two genomes over other social traits beyond suicide, then there may be further reasons to expect genomic imprinting (and potentially of both maternal‐origin and paternal‐origin genes). Furthermore, although not considered in the present analysis, we might expect parents to disagree with offspring over the social traits that they should express. In particular, mothers in PGE species may be expected to favor lower levels of altruism than the male paternal‐origin genome in their sons, and thus they may be favored to silence this genome if possible. Moreover, if sons preferentially direct their altruism to female kin, then monogeny (seen in both sciarid flies and gall midges [Hodson and Ross 2021]) may be a further mechanism to reduce such altruistic behavior in sons. This array of intergenomic and intragenomic conflict of interests that PGE generates may provide an explanation for not only the remarkable diversity of genetic systems in these groups, but also the dynamic transitions between them (Ross et al. 2010).

## CONFLICT OF INTEREST

The authors declare no conflict of interest.

## AUTHOR CONTRIBUTIONS

TJH and AG jointly designed the study. TJH performed the analysis. TJH and AG wrote the manuscript.

## DATA ARCHIVING

There are no data to be archived.

Associate Editor: Dr. B. Kuijper

Handling Editor: Dr. A. McAdam

## Supporting information

Supplementary Material
