# Supplementary material for: Paternal genome elimination promotes altruism in viscous populations
Source: Evolution. 2022 Aug 7;76(9):2191–8. doi: 10.1111/evo.14585 (PMC9543263; doi:10.1111/evo.14585)
Supplement: Supplementary file 1 — Supplementary Material [file EVO-76-2191-s001.pdf]

Supplementary Material for:
Paternal genome elimination promotes altruism in viscous populations

Thomas J. Hitchcock[1*] and Andy Gardner[1]

[1]School of Biology, University of St Andrews, St Andrews, UK

[*]Correspondence: th76@st-andrews.ac.uk

1 Life cycle

Here we analyse the following life-cycle, illustrated in Figure S1. This is a more general version than that described in the main text, allowing for various sexual asymmetries in ecology, such as dispersal and adult sex ratio. We assume that there is a infinite population subdivided into a large number of patches. It proceeds as so: (1) a large number of juveniles are born onto a patch, of which a proportion ρ are male and $1-\rho$ female. (2) Individuals then engage in a social behaviour z_j which modulates their survival to adulthood, which is S_f for females and S_m for males. (3) Individuals then disperse from their natal patch with sex-specific probabilities, d_f for females and d_m for males. (4) Post-dispersal, individuals compete for the n_f female and n_m male breeding spots on each patch, unsuccessful individuals die. (5) Adult individuals then engage in a second social behaviour z_a which modulates their fecundity F_f for females and F_m for males. After producing new juveniles, the adults on the patch die, and the life-cycle begins once more.

2 Fitness functions

In our model there two classes of individual, female and male, and four possible types of transition between these two classes (females through daughters, females through sons, males through daughters, males through sons). The absolute fitness of a parent of class $\mathbb{i}$ through production of class $\mathbb{j}$ offspring is written as $w_{\mathbb{i}\to\mathbb{j}}$. We census the population at phase (1) of the life cycle described above, and thus $w_{\mathbb{i}\to\mathbb{j}}$ refers to the absolute number of new individuals of class $\mathbb{j}$ in the next iteration of the life cycle to whom this focal individual is assigned parentage. The relative fitness of an individual is their absolute fitness through a particular route in the life cycle divided by the absolute fitness of an average individual through that same class transition. This can be written as:

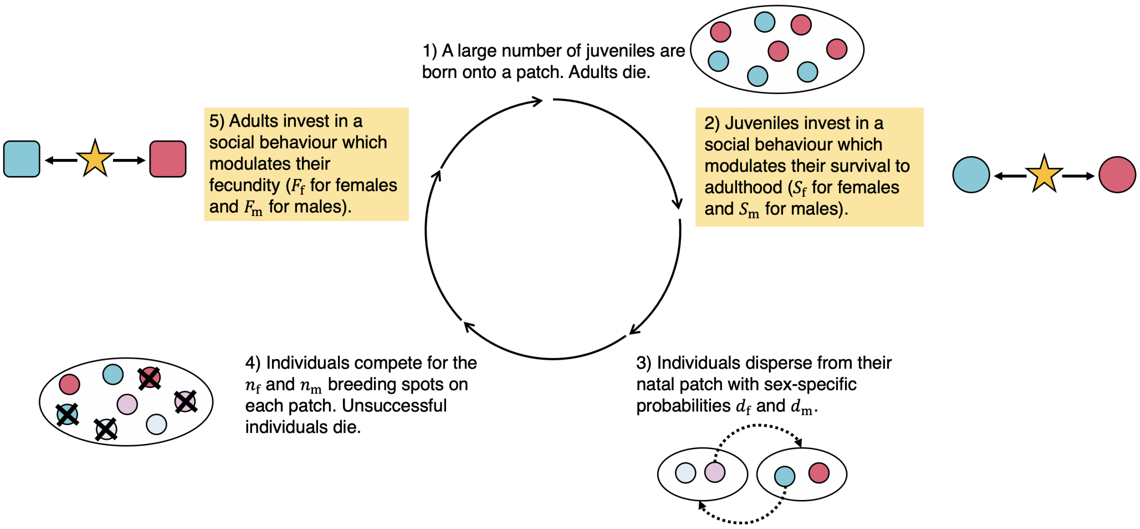

Figure S1: **Description of the key phases in the life-cycle.** These include: birth (1), juvenile social behaviour and survival (2), dispersal (3), competition for breeding spots (4), and adult social behaviour (5). The two phases highlighted in yellow represent the stages in which our two social behaviours analysed (z_j and z_a) occur.

$$W_{\mathbb{i}\to\mathbb{j}} = \frac{w_{\mathbb{i}\to\mathbb{j}}}{\overline{w}_{\mathbb{i}\to\mathbb{j}}} \tag{S1}$$

The absolute fitness of an individual of sex $\mathbb{i}$ through sex $\mathbb{j}$ offspring $w_{\mathbb{i}\to\mathbb{j}}$ in our life cycle is the product of: their probability of survival $S_{\mathbb{i}}$ (phase 2), the probability they obtain a breeding spot $\theta_{\mathbb{i}}$ (phase 3), and their fecundity $F_{\mathbb{i}}$ (phase 5). We can write these out as so:

$$w_{f\to f} = S_f \times \theta_f \times F_f(1-\rho) \tag{S2a}$$

$$w_{f\to m} = S_f \times \theta_f \times F_f\rho \tag{S2b}$$

$$w_{m\to f} = S_m \times \theta_m \times F_m(1-\rho) \tag{S2c}$$

$$w_{m\to m} = S_m \times \theta_m \times F_m\rho \tag{S2d}$$

A focal individual's survival is a function of both their own juvenile trait value $x_{\mathbb{i}j}$, and the trait value of their juvenile female y_{fj} and male social partners y_{mj}, e.g. a focal female's survival is $S_f\left(x_{fj}, y_{fj}, y_{mj}\right)$. A focal individual's fecundity is a function of their own adult trait value $x_{\mathbb{i}a}$ and the average female y_{fa} and male trait value y_{ma} in their focal patch (including themselves), e.g. a focal female's fecundity is $F_f\left(x_{fa}, y_{fa}, y_{ma}\right)$. We allow these social behaviours to be sex-limited in their expression, in these cases only the sex which expresses the trait will impact these phenotypes.

The probabilities that a focal female θ_f and a focal male θ_m obtain a breeding spot are not directly functions of the focal individual's trait value (i.e. competition is random with respect to phenotype), but instead is a function of the number of same sex competitors on their patch, which is determined by: the average fecundities of

the adult females and males on the focal patch in the previous generation $\hat{F}_{\mathrm{f}}$ and $\hat{F}_{\mathrm{m}}$, the average fecundities in the population $\overline{F}_{\mathrm{f}}$ and $\overline{F}_{\mathrm{m}}$, the average survival of same sex juveniles on the focal patch $\hat{S}_{\mathrm{f}}$ and $\hat{S}_{\mathrm{m}}$, and the survival probabilities of juveniles on the average patch in the population $\overline{S}_{\mathrm{f}}$ and $\overline{S}_{\mathrm{m}}$. This can be written out as so:

$$\theta_{\mathrm{f}} = \frac{(1-d_{\mathrm{f}})n_{\mathrm{f}}}{(1-d_{\mathrm{f}})n_{\mathrm{f}}\hat{F}_{\mathrm{f}}(1-\rho)\hat{S}_{\mathrm{f}} + d_{\mathrm{f}}n_{\mathrm{f}}\overline{F}_{\mathrm{f}}(1-\rho)\overline{S}_{\mathrm{f}}} + \frac{d_{\mathrm{f}}n_{\mathrm{f}}}{n_{\mathrm{f}}\overline{F}_{\mathrm{f}}(1-\rho)\overline{S}_{\mathrm{f}}} \tag{S3a}$$

$$\theta_{\mathrm{m}} = \frac{(1-d_{\mathrm{m}})n_{\mathrm{m}}}{(1-d_{\mathrm{m}})n_{\mathrm{m}}\hat{F}_{\mathrm{m}}\rho\hat{S}_{\mathrm{m}} + d_{\mathrm{m}}n_{\mathrm{m}}\overline{F}_{\mathrm{m}}\rho\overline{S}_{\mathrm{m}}} + \frac{d_{\mathrm{m}}n_{\mathrm{m}}}{n_{\mathrm{m}}\overline{F}_{\mathrm{m}}\rho\overline{S}_{\mathrm{m}}} \tag{S3b}$$

In addition, note the constraint that $n_{\mathrm{f}}\hat{F}_{\mathrm{f}} = n_{\mathrm{m}}\hat{F}_{\mathrm{m}}$ and $n_{\mathrm{f}}\overline{F}_{\mathrm{f}} = n_{\mathrm{m}}\overline{F}_{\mathrm{m}}$. We do not assume whether it is males or females who ultimately are the limiting factor upon reproduction and thus determine the fecundity of the patch (and population). We simply place the constraint that the total fecundity of males and females on each patch (and in the population) is equal. Subsequently, it is possible to interchange these fecundities in the fitness expressions. This is similar to the approach used by Johnstone and Cant (2008). Converting our expressions for absolute fitness to relative fitness, they become:

$$W_{\mathrm{f}\to\mathrm{f}} = \frac{w_{\mathrm{f}\to\mathrm{f}}}{\overline{w}_{\mathrm{f}\to\mathrm{f}}} = (1-d_{\mathrm{f}})\frac{S_{\mathrm{f}}F_{\mathrm{f}}}{(1-d_{\mathrm{f}})\hat{S}_{\mathrm{f}}\hat{F}_{\mathrm{f}} + d_{\mathrm{f}}\overline{S}_{\mathrm{f}}\overline{F}_{\mathrm{f}}} + d_{\mathrm{f}}\frac{S_{\mathrm{f}}F_{\mathrm{f}}}{\overline{S}_{\mathrm{f}}\overline{F}_{\mathrm{f}}} \tag{S4a}$$

$$W_{\mathrm{f}\to\mathrm{m}} = \frac{w_{\mathrm{f}\to\mathrm{m}}}{\overline{w}_{\mathrm{f}\to\mathrm{m}}} = (1-d_{\mathrm{f}})\frac{S_{\mathrm{f}}F_{\mathrm{f}}}{(1-d_{\mathrm{f}})\hat{S}_{\mathrm{f}}\hat{F}_{\mathrm{f}} + d_{\mathrm{f}}\overline{S}_{\mathrm{f}}\overline{F}_{\mathrm{f}}} + d_{\mathrm{f}}\frac{S_{\mathrm{f}}F_{\mathrm{f}}}{\overline{S}_{\mathrm{f}}\overline{F}_{\mathrm{f}}} \tag{S4b}$$

$$W_{\mathrm{m}\to\mathrm{f}} = \frac{w_{\mathrm{m}\to\mathrm{f}}}{\overline{w}_{\mathrm{m}\to\mathrm{f}}} = (1-d_{\mathrm{m}})\frac{S_{\mathrm{m}}F_{\mathrm{m}}}{(1-d_{\mathrm{m}})\hat{S}_{\mathrm{m}}\hat{F}_{\mathrm{m}} + d_{\mathrm{m}}\overline{S}_{\mathrm{m}}\overline{F}_{\mathrm{m}}} + d_{\mathrm{m}}\frac{S_{\mathrm{m}}F_{\mathrm{m}}}{\overline{S}_{\mathrm{m}}\overline{F}_{\mathrm{m}}} \tag{S4c}$$

$$W_{\mathrm{m}\to\mathrm{m}} = \frac{w_{\mathrm{m}\to\mathrm{m}}}{\overline{w}_{\mathrm{m}\to\mathrm{m}}} = (1-d_{\mathrm{m}})\frac{S_{\mathrm{m}}F_{\mathrm{m}}}{(1-d_{\mathrm{m}})\hat{S}_{\mathrm{m}}\hat{F}_{\mathrm{m}} + d_{\mathrm{m}}\overline{S}_{\mathrm{m}}\overline{F}_{\mathrm{m}}} + d_{\mathrm{m}}\frac{S_{\mathrm{m}}F_{\mathrm{m}}}{\overline{S}_{\mathrm{m}}\overline{F}_{\mathrm{m}}} \tag{S4d}$$

3 Marginal fitness effects

We now calculate the marginal fitness effects associated with a small change in the behaviour of different groups of individual upon our focal individuals. We notate the trait value of a focal individual of sex $\Bbbk$ and of locus $\mathbb{l}$, $x_{\Bbbk\mathbb{l}}$, and of the average group member as $y_{\Bbbk\mathbb{l}}$, and of the average group member in the parents generation as $Y_{\Bbbk\mathbb{l}}$. We make the following substitutions.

For our juvenile behaviours:

$$\frac{\partial(S_{\mathrm{f}}/\overline{S}_{\mathrm{f}})}{\partial x_{\mathrm{fj}}} = -c_{\mathrm{fj}} \tag{S5a}$$

$$\frac{\partial(S_{\mathrm{m}}/\overline{S}_{\mathrm{m}})}{\partial x_{\mathrm{mj}}} = -c_{\mathrm{mj}} \tag{S5b}$$

$$\frac{\partial(S_{\mathrm{f}}/\overline{S}_{\mathrm{f}})}{\partial y_{\mathrm{fj}}} = \frac{\partial(S_{\mathrm{m}}/\overline{S}_{\mathrm{m}})}{\partial y_{\mathrm{fj}}} = b_{\mathrm{fj}} \tag{S5c}$$

$$\frac{\partial(S_{\mathrm{f}}/\overline{S}_{\mathrm{f}})}{\partial y_{\mathrm{mj}}} = \frac{\partial(S_{\mathrm{m}}/\overline{S}_{\mathrm{m}})}{\partial y_{\mathrm{mj}}} = b_{\mathrm{mj}} \tag{S5d}$$

	∂x_{fj}	∂y_{fj}	∂x_{mj}	∂y_{mj}
∂W_{ff}	$-c_{fj}$	$b_{fj} - \kappa_f(b_{fj} - c_{fj})$	0	$b_{mj}(1 - \kappa_f)$
∂W_{fm}	$-c_{fj}$	$b_{fj} - \kappa_f(b_{fj} - c_{fj})$	0	$b_{mj}(1 - \kappa_f)$
∂W_{mf}	0	$b_{fj}(1 - \kappa_m)$	$-c_{mj}$	$b_{mj} - \kappa_m(b_{mj} - c_{mj})$
∂W_{mm}	0	$b_{fj}(1 - \kappa_m)$	$-c_{mj}$	$b_{mj} - \kappa_m(b_{mj} - c_{mj})$

Table S1: Marginal fitness effects for different genetic actors on self and social partners, for our juvenile trait affecting survival, where $\kappa_f = (1 - d_f)^2$ and $\kappa_m = (1 - d_m)^2$.

	∂x_{fa}	∂y_{fa}	∂Y_{fa}	∂x_{ma}	∂y_{ma}	∂Y_{ma}
∂W_{ff}	$-c_{fa}$	b_{fa}	$-\kappa_f(b_{fa} - c_{fa})$	0	$\frac{n_m}{n_f}(b_{ma} - c_{ma})$	$-\frac{n_m}{n_f}\kappa_f(b_{ma} - c_{ma})$
∂W_{fm}	$-c_{fa}$	b_{fa}	$-\kappa_f(b_{fa} - c_{fa})$	0	$\frac{n_m}{n_f}(b_{ma} - c_{ma})$	$-\frac{n_m}{n_f}\kappa_f(b_{ma} - c_{ma})$
∂W_{mf}	0	$\frac{n_f}{n_m}(b_{fa} - c_{fa})$	$-\frac{n_f}{n_m}\kappa_m(b_{fa} - c_{fa})$	$-c_{ma}$	b_{ma}	$-\kappa_m(b_{ma} - c_{ma})$
∂W_{mm}	0	$\frac{n_f}{n_m}(b_{fa} - c_{fa})$	$-\frac{n_f}{n_m}\kappa_m(b_{fa} - c_{fa})$	$-c_{ma}$	b_{ma}	$-\kappa_m(b_{ma} - c_{ma})$

Table S2: Marginal fitness effects for different genetic actors on self and social partners for our adult trait affecting fecundity, where $\kappa_f = (1 - d_f)^2$ and $\kappa_m = (1 - d_m)^2$.

And for our adult behaviours:

$$\frac{\partial(F_f/\overline{F}_f)}{\partial x_{fa}} = -c_{fa} \tag{S6a}$$

$$\frac{\partial(F_m/\overline{F}_m)}{\partial x_{ma}} = -c_{ma} \tag{S6b}$$

$$\frac{\partial(F_f/\overline{F}_f)}{\partial y_{fa}} = b_{fa} \tag{S6c}$$

$$\frac{\partial(F_m/\overline{F}_m)}{\partial y_{fa}} = \frac{n_f}{n_m}(b_{fa} - c_{fa}) \tag{S6d}$$

$$\frac{\partial(F_f/\overline{F}_f)}{\partial y_{ma}} = \frac{n_m}{n_f}(b_{ma} - c_{ma}) \tag{S6e}$$

$$\frac{\partial(F_m/\overline{F}_m)}{\partial y_{ma}} = b_{ma} \tag{S6f}$$

Making these substitutions, we can see the marginal fitness effects associated with a change in different social partners. In Table 1, we can see the marginal fitness effects associated with a change in the value of our juvenile social behaviours. In Table 2, we can see the marginal fitness effects associated with a change in the value of our adult social behaviours.

4 Consanguinities and relatedness coefficients

In order to calculate the relatedness coefficients between our different individuals, we first calculate the consanguinities between our different gene positions. These, in turn, are calculated by writing out recursions to

describe the probability of identity by descent between our different sets of gene positions in a neutral population. Assuming that the consanguinity coefficients have obtained their quasi-equilibrium values, such that the consanguinity between two gene positions in the next generation are equal to the consanguinity between those same two gene positions in this generation i.e. $Q'_{\mathbb{x},\mathbb{y}} = Q_{\mathbb{x},\mathbb{y}}$, then we may write out a system of simultaneous equations, which we can then solve in terms of our demographic parameters. Note that these are approximations of the true consanguinities, as genealogies may be altered by the action of selection, but is reasonable provided selection is weak (Frank, 1998; Rousset, 2004; Gardner et al., 2011).

Notation proceeds as follows. We notate the consanguinity between two genes sampled within an individual (with replacement) as $Q^i_{\mathbb{x},\mathbb{y}}$, between two juvenile individuals on a patch without replacement as $Q^p_{\mathbb{x},\mathbb{y}}$, and between a juvenile $\mathbb{x}$ in the current generation and an adult $\mathbb{y}$ on the same patch in the previous generation as $Q^v_{\mathbb{x},\mathbb{y}}$. For the haploid case, we have simply two types of gene position - males m and females f. In the diploid case, we have four gene positions: female maternal-origin genes $\mathrm{f_M}$, female paternal-origin genes $\mathrm{f_P}$, male maternal-origin genes $\mathrm{m_M}$, and male paternal-origin genes $\mathrm{m_P}$. So, for example, the probability of identity by descent between a female maternal-origin gene and a male paternal-origin gene from two juveniles on the same patch, would be written as $Q^p_{\mathrm{f_M},\mathrm{m_P}}$.

To describe the genetical system, we notate the probability that a maternal-origin gene was inherited from a maternal-origin gene α, and the probability a paternal-origin gene was inherited from a paternal-origin gene β. For the haploid case we have λ, which is the probability that a gene (in either a male or female) was inherited from a female. These parameters allows us to capture our various inheritance systems of interest in a single set of equations. For 'eumendelian' diploidy we have $\alpha = 1/2$,and $\beta = 1/2$, for arrhenotoky and male PGE we have $\alpha = 1/2$ and $\beta = 0$, and for paterothylotoky and female MGE we have $\alpha = 0$ and $\beta = 1/2$. For the haploid case, it allows us to explore the full range from full matrilineal inheritance $\lambda = 1$, to full patrilineal inheritance $\lambda = 0$.

To describe the mating system, we notate the probability that two juveniles born on the same patch share a mother $\mathscr{A}$, and the probability that two juveniles born on the same patch share a father $\mathscr{B}$. For our analysis we assume that $\mathscr{A} = 1/n_\mathrm{f}$ and $\mathscr{B} = 1/n_\mathrm{m}$. However, we write this out in the more general form here to draw similarities to other analyses (e.g. Gardner, 2010). The probability of inbreeding, i.e. the probability that two individuals born on the same patch mate, is given by $\phi = (1-d_\mathrm{f})(1-d_\mathrm{m})$.

Haploidy

Within individuals

In the haploid case, the probability that two gene copies sampled within an individual (with replacement) are identical by descent (IBD) is simply 1:

$$Q^i_{\mathrm{f,f}} = Q^i_{\mathrm{m,m}} = 1 \tag{S7a}$$

Between juvenile patchmates

The probability that two genes sampled in different juvenile patchmates are IBD is the probability that either they come from the same sex parent (λ^2 or $(1-\lambda)^2$), in which case they are IBD if they come from the same parent ($\mathscr{A}$ or $\mathscr{B}$), or if they come from different parents ($1-\mathscr{A}$ or $1-\mathscr{B}$) then they are IBD with the probability that those parents were natal to the patch and are IBD ($(1-d_\mathrm{f})^2 Q^p_{\mathrm{f,f}}$ or $(1-d_\mathrm{m})^2 Q^p_{\mathrm{m,m}}$). Alternatively, if they come from different sex parents, then they are IBD if those parents are both natal to the patch (ϕ), and were IBD as juveniles ($Q^p_{\mathrm{f,m}}$).

$$\begin{aligned} Q^p_{\mathrm{f,f}} = Q^p_{\mathrm{f,m}} = Q^p_{\mathrm{m,f}} = Q^p_{\mathrm{m,m}} = \\ \lambda\left(\lambda\left(\mathscr{A}Q^i_{\mathrm{f,f}} + (1-\mathscr{A})(1-d_\mathrm{f})^2 Q^p_{\mathrm{f,f}}\right) + (1-\lambda)\phi Q^p_{\mathrm{f,m}}\right) \\ + (1-\lambda)\left(\lambda\phi Q^p_{\mathrm{f,m}} + (1-\lambda)\left(\mathscr{B}Q^i_{\mathrm{m,m}} + (1-\mathscr{B})(1-d_\mathrm{m})^2 Q^p_{\mathrm{m,m}}\right)\right) \end{aligned} \tag{S8a}$$

Between generations

Genes sampled in a juvenile and an adult female are IBD if first the gene in the juvenile came from a female in the previous generation (λ), if so then either it came directly from that female ($\mathscr{A}$), or if it came from another female ($1-\mathscr{A}$), in which case they are IBD if those two females were both natal to the patch ($(1-d_\mathrm{f})^2$), and were IBD as juveniles ($Q^p_{\mathrm{f,f}}$). If the gene sampled in a juvenile came from a male ($1-\lambda$), then it would be the probability that the adult female and that father were both natal to the patch (ϕ), and were IBD as juveniles ($Q^p_{\mathrm{f,m}}$.) A similar logic can be used to calculate the consanguinity between a juvenile and an adult male.

$$Q^v_{\mathrm{f,f}} = Q^v_{\mathrm{m,f}} = \lambda\left(\mathscr{A}Q^i_{\mathrm{f,f}} + (1-\mathscr{A})(1-d_\mathrm{f})^2 Q^p_{\mathrm{f,f}}\right) + (1-\lambda)\phi Q^p_{\mathrm{f,m}} \tag{S9a}$$

$$Q^v_{\mathrm{f,m}} = Q^v_{\mathrm{m,m}} = \lambda\phi Q^p_{\mathrm{f,m}} + (1-\lambda)\left(\mathscr{B}Q^i_{\mathrm{m,m}} + (1-\mathscr{B})(1-d_\mathrm{m})^2 Q^p_{\mathrm{m,m}}\right) \tag{S9b}$$

Diploidy

The equations for diploidy follow a similar logic to those for haploidy, except we now have additional gene positions ($\mathrm{f_M}, \mathrm{f_P}, \mathrm{m_M}, \mathrm{m_P}$), and additional notation to describe the transmission genetics (α, β).

Within individuals

$$Q^i_{\mathrm{f_M,f_M}} = Q^i_{\mathrm{f_P,f_P}} = Q^i_{\mathrm{m_M,m_M}} = Q^i_{\mathrm{m_P,m_P}} = 1 \tag{S10a}$$

$$Q^i_{\mathrm{f_M,f_P}} = Q^i_{\mathrm{m_M,m_P}} = \alpha\left((1-\beta)\phi Q^p_{\mathrm{f_M,m_M}} + \beta\phi Q^p_{\mathrm{f_M,m_P}}\right) + (1-\alpha)\left((1-\beta)\phi Q^p_{\mathrm{f_P,m_M}} + \beta\phi Q^p_{\mathrm{f_P,m_P}}\right) \tag{S10b}$$

Between juvenile patchmates

$$\begin{aligned} Q^p_{\mathrm{f_M,f_M}} = \alpha\left(\alpha\left(\mathscr{A}Q^i_{\mathrm{f_M,f_M}} + (1-\mathscr{A})(1-d_\mathrm{f})^2 Q^p_{\mathrm{f_M,f_M}}\right) + (1-\alpha)\left(\mathscr{A}Q^i_{\mathrm{f_M,f_P}} + (1-\mathscr{A})(1-d_\mathrm{f})^2 Q^p_{\mathrm{f_M,f_P}}\right)\right) \\ + (1-\alpha)\left(\alpha\left(\mathscr{A}Q^i_{\mathrm{f_M,f_P}} + (1-\mathscr{A})(1-d_\mathrm{f})^2 Q^p_{\mathrm{f_M,f_P}}\right) + (1-\alpha)\left(\mathscr{A}Q^i_{\mathrm{f_P,f_P}} + (1-\mathscr{A})(1-d_\mathrm{f})^2 Q^p_{\mathrm{f_P,f_P}}\right)\right) \end{aligned} \tag{S11a}$$

$$
\begin{aligned}
Q^p_{\mathrm{f_M,f_P}} &= \alpha\left((1-\beta)\phi Q^p_{\mathrm{f_M,m_M}} + \beta\phi Q^p_{\mathrm{f_M,m_P}}\right)\\
&+ (1-\alpha)\left((1-\beta)\phi Q^p_{\mathrm{f_P,m_M}} + \beta\phi Q^p_{\mathrm{f_P,m_P}}\right)
\end{aligned} \tag{S11b}
$$

$$
\begin{aligned}
Q^p_{\mathrm{f_P,f_P}} = (1-\beta)&\left((1-\beta)\left(\mathscr{B}Q^i_{\mathrm{m_M,m_M}} + (1-\mathscr{B})(1-d_\mathrm{m})^2 Q^p_{\mathrm{m_M,m_M}}\right) + \beta\left(\mathscr{B}Q^i_{\mathrm{m_M,m_P}} + (1-\mathscr{B})(1-d_\mathrm{m})^2 Q^p_{\mathrm{m_M,m_P}}\right)\right)\\
&+ \beta\left((1-\beta)\left(\mathscr{B}Q^i_{\mathrm{m_M,m_P}} + (1-\mathscr{B})(1-d_\mathrm{m})^2 Q^p_{\mathrm{m_M,m_P}} +\right) + \beta\left(\mathscr{B}Q^i_{\mathrm{m_P,m_P}} + (1-\mathscr{B})(1-d_\mathrm{m})^2 Q^p_{\mathrm{m_P,m_P}}\right)\right)
\end{aligned} \tag{S11c}
$$

$$
\begin{aligned}
Q^p_{\mathrm{f_M,m_M}} &= \alpha\left(\alpha\left(\mathscr{A}Q^i_{\mathrm{f_M,f_M}} + (1-\mathscr{A})(1-d_\mathrm{f})^2 Q^p_{\mathrm{f_M,f_M}}\right) + (1-\alpha)\left(\mathscr{A}Q^i_{\mathrm{f_M,f_P}} + (1-\mathscr{A})(1-d_\mathrm{f})^2 Q^p_{\mathrm{f_M,f_P}}\right)\right)\\
&+ (1-\alpha)\left(\alpha\left(\mathscr{A}Q^i_{\mathrm{f_M,f_P}} + (1-\mathscr{A})(1-d_\mathrm{f})^2 Q^p_{\mathrm{f_M,f_P}}\right) + (1-\alpha)\left(\mathscr{A}Q^i_{\mathrm{f_P,f_P}} + (1-\mathscr{A})(1-d_\mathrm{f})^2 Q^p_{\mathrm{f_P,f_P}}\right)\right)
\end{aligned} \tag{S11d}
$$

$$
\begin{aligned}
Q^p_{\mathrm{f_M,m_P}} &= \alpha\left((1-\beta)\phi Q^p_{\mathrm{f_M,m_M}} + \beta\phi Q^p_{\mathrm{f_M,m_P}}\right)\\
&+ (1-\alpha)\left((1-\beta)\phi Q^p_{\mathrm{f_P,m_M}} + \beta\phi Q^p_{\mathrm{f_P,m_P}}\right)
\end{aligned} \tag{S11e}
$$

$$
\begin{aligned}
Q^p_{\mathrm{f_P,m_P}} = (1-\beta)&\left((1-\beta)\left(\mathscr{B}Q^i_{\mathrm{m_M,m_M}} + (1-\mathscr{B})(1-d_\mathrm{m})^2 Q^p_{\mathrm{m_M,m_M}}\right) + \beta\left(\mathscr{B}Q^i_{\mathrm{m_M,m_P}} + (1-\mathscr{B})(1-d_\mathrm{m})^2 Q^p_{\mathrm{m_M,m_P}}\right)\right)\\
&+ \beta\left((1-\beta)\left(\mathscr{B}Q^i_{\mathrm{m_M,m_P}} + (1-\mathscr{B})(1-d_\mathrm{m})^2 Q^p_{\mathrm{m_M,m_P}}\right) + \beta\left(\mathscr{B}Q^i_{\mathrm{m_P,m_P}} + (1-\mathscr{B})(1-d_\mathrm{m})^2 Q^p_{\mathrm{m_P,m_P}}\right)\right)
\end{aligned} \tag{S11f}
$$

$$
\begin{aligned}
Q^p_{\mathrm{m_M,m_M}} &= \alpha\left(\alpha\left(\mathscr{A}Q^i_{\mathrm{f_M,f_M}} + (1-\mathscr{A})(1-d_\mathrm{f})^2 Q^p_{\mathrm{f_M,f_M}}\right) + (1-\alpha)\left(\mathscr{A}Q^i_{\mathrm{f_M,f_P}} + (1-\mathscr{A})(1-d_\mathrm{f})^2 Q^p_{\mathrm{f_M,f_P}}\right)\right)\\
&+ (1-\alpha)\left(\alpha\left(\mathscr{A}Q^i_{\mathrm{f_M,f_P}} + (1-\mathscr{A})(1-d_\mathrm{f})^2 Q^p_{\mathrm{f_M,f_P}}\right) + (1-\alpha)\left(\mathscr{A}Q^i_{\mathrm{f_P,f_P}} + (1-\mathscr{A})(1-d_\mathrm{f})^2 Q^p_{\mathrm{f_P,f_P}}\right)\right)
\end{aligned} \tag{S11g}
$$

$$
\begin{aligned}
Q^p_{\mathrm{m_M,m_P}} &= \alpha\left((1-\beta)\phi Q^p_{\mathrm{f_M,m_M}} + \beta\phi Q^p_{\mathrm{f_M,m_P}}\right)\\
&+ (1-\alpha)\left((1-\beta)\phi Q^p_{\mathrm{f_P,m_M}} + \beta\phi Q^p_{\mathrm{f_P,m_P}}\right)
\end{aligned} \tag{S11h}
$$

$$
\begin{aligned}
Q^p_{\mathrm{m_P,m_P}} = (1-\beta)&\left((1-\beta)\left(\mathscr{B}Q^i_{\mathrm{m_M,m_M}} + (1-\mathscr{B})(1-d_\mathrm{m})^2 Q^p_{\mathrm{m_M,m_M}}\right) + \beta\left(\mathscr{B}Q^i_{\mathrm{m_M,m_P}} + (1-\mathscr{B})(1-d_\mathrm{m})^2 Q^p_{\mathrm{m_M,m_P}}\right)\right)\\
&+ \beta\left((1-\beta)\left(\mathscr{B}Q^i_{\mathrm{m_M,m_P}} + (1-\mathscr{B})(1-d_\mathrm{m})^2 Q^p_{\mathrm{m_M,m_P}} +\right) + \beta\left(\mathscr{B}Q^i_{\mathrm{m_P,m_P}} + (1-\mathscr{B})(1-d_\mathrm{m})^2 Q^p_{\mathrm{m_P,m_P}}\right)\right)
\end{aligned} \tag{S11i}
$$

Between generations

$$
Q^v_{\mathrm{f_M,f_M}} = \alpha\left(\mathscr{A}Q^i_{\mathrm{f_M,f_M}} + (1-\mathscr{A})(1-d_\mathrm{f})^2 Q^p_{\mathrm{f_M,f_M}}\right) + (1-\alpha)\left(\mathscr{A}Q^i_{\mathrm{f_M,f_P}} + (1-\mathscr{A})(1-d_\mathrm{f})^2 Q^p_{\mathrm{f_M,f_P}}\right) \tag{S12a}
$$

$$
Q^v_{\mathrm{f_M,f_P}} = \alpha\left(\mathscr{A}Q^i_{\mathrm{f_M,f_P}} + (1-\mathscr{A})(1-d_\mathrm{f})^2 Q^p_{\mathrm{f_M,f_P}}\right) + (1-\alpha)\left(\mathscr{A}Q^i_{\mathrm{f_P,f_P}} + (1-\mathscr{A})(1-d_\mathrm{f})^2 Q^p_{\mathrm{f_P,f_P}}\right) \tag{S12b}
$$

$$
Q^v_{\mathrm{f_M,m_M}} = \alpha\phi Q^p_{\mathrm{f_M,m_M}} + (1-\alpha)\phi Q^p_{\mathrm{f_P,m_M}} \tag{S12c}
$$

$$Q^{v}_{\mathrm{f_M},\mathrm{m_P}} = \alpha\phi Q^{p}_{\mathrm{f_M},\mathrm{m_P}} + (1-\alpha)\phi Q^{p}_{\mathrm{f_P},\mathrm{m_P}} \tag{S12d}$$

$$Q^{v}_{\mathrm{f_P},\mathrm{f_M}} = (1-\beta)\phi Q^{p}_{\mathrm{f_M},\mathrm{m_M}} + \beta\phi Q^{p}_{\mathrm{f_M},\mathrm{m_P}} \tag{S13a}$$

$$Q^{v}_{\mathrm{f_P},\mathrm{f_P}} = (1-\beta)\phi Q^{p}_{\mathrm{f_P},\mathrm{m_M}} + \beta\phi Q^{p}_{\mathrm{m_P},\mathrm{m_P}} \tag{S13b}$$

$$Q^{v}_{\mathrm{f_P},\mathrm{m_M}} = (1-\beta)\left(\mathscr{B}Q^{i}_{\mathrm{m_M},\mathrm{m_M}} + (1-\mathscr{B})(1-d_{\mathrm{m}})^2 Q^{p}_{\mathrm{m_M},\mathrm{m_M}}\right) + \beta\left(\mathscr{B}Q^{i}_{\mathrm{m_M},\mathrm{m_P}} + (1-\mathscr{B})(1-d_{\mathrm{m}})^2 Q^{p}_{\mathrm{m_M},\mathrm{m_P}}\right) \tag{S13c}$$

$$Q^{v}_{\mathrm{f_P},\mathrm{m_P}} = (1-\beta)\left(\mathscr{B}Q^{i}_{\mathrm{m_M},\mathrm{m_P}} + (1-\mathscr{B})(1-d_{\mathrm{m}})^2 Q^{p}_{\mathrm{m_M},\mathrm{m_P}}\right) + \beta\left(\mathscr{B}Q^{i}_{\mathrm{m_P},\mathrm{m_P}} + (1-\mathscr{B})(1-d_{\mathrm{m}})^2 Q^{p}_{\mathrm{m_P},\mathrm{m_P}}\right) \tag{S13d}$$

Relatedness coefficients

We can now calculate the relatedness coefficients as a weighted sum of the above consanguinity coefficients. Such weightings are necessary in this case because - with paternal-genome elimination - not all of the genes within an individual have the same prospects going forward. As we are performing a personal fitness analysis, then the required relatedness coefficients $r_{\mathbb{x},\mathbb{y}}$ describe the correlation between a focal individual's transmitted breeding value, which we denote $g_{\mathbb{x}}$, and the somatic breeding value of their social partners (including themselves), which we denote $G_{\mathbb{y}}$. Thus, the consanguinities between the gene copies within an individual to those in their social partners must be weighted in proportion to their contribution to the transmitted breeding value. Note, however, that these relatedness coefficients are distinct from those used in an inclusive fitness analysis (see Frank, 1998, Chapter 4), where instead relatedness is the correlation between a focal individual's somatic breeding value and their social partners' transmitted breeding value.

Similar to above, we denote the somatic breeding value of our focal individual G^{i}, the somatic breeding value of a juvenile individual on the focal patch as G^{p}, the somatic breeding value of an adult individual on the focal patch (including oneself) as G^{q}, and the somatic breeding value of an adult individual in the focal patch in the previous generation as G^{v}.

Earlier, we denoted the probability that a maternal-origin gene came from a maternal-origin gene α, and similarly we denoted the probability that a paternal-origin gene came from a paternal-origin gene β. Let the contribution of a female's maternal-origin gene to her transmitted breeding value be $\hat{\alpha}$, and the contribution of a male's paternal-origin gene to his transmitted breeding value be $\hat{\beta}$. In the case of this model, as the probability that maternal-origin gene came from a maternal-origin gene is α and the probability it comes from a paternal-origin gene $1-\alpha$, then the contribution that the maternal-origin makes to the transmitted breeding value of a female is simply $\hat{\alpha} = \alpha$, and similarly for the contribution of the paternal-origin gene to the transmitted breeding value of a male $\hat{\beta} = \beta$.

To allow for differential contributions to the somatic breeding value (i.e. the expressed phenotype), we denote σ to be the fraction of a female's somatic breeding value that comes from the her maternal-origin gene copy, and τ to be the fraction of a male's somatic breeding value that comes from his paternal-origin gene copy. For example, in the case of paternal genome elimination $\alpha = 1/2, \beta = 0$, if all gene copies were expressed then $\sigma = 1/2, \tau = 1/2$, whilst if the male paternal genome is silenced then $\sigma = 1/2, \tau = 0$, and if the male maternal genome is silenced then $\sigma = 1/2, \tau = 1$. These two parameters thus allow us to manipulate the degree of "control" that the maternal-origin and paternal-origin genes exert over the phenotype in females and males, biologically this would most likely arise from parent-of-origin specific gene expression, e.g. imprinting. Moreover, these tools allows us to investigate genetic systems such as classical haplodiploidy (e.g. arrhenotoky) within a diploid genetic system, by ignoring the contribution of the paternal-origin genome in males to the phenotype (i.e. by setting $\tau = 0$). For arrhenotoky and pareothylotoky, we additionally assume $\sigma = 1/2, \tau = 0$ and $\sigma = 0, \tau = 1/2$ respectively. For standard diploid we assume $\sigma = 1/2, \tau = 1/2$.

Haploidy

Within individuals

$$\frac{\mathrm{d}G_{\mathrm{f}}^{i}}{\mathrm{d}g_{\mathrm{f}}} = r_{\mathrm{f,f}}^{i} = Q_{\mathrm{f,f}}^{i} \tag{S14a}$$

$$\frac{\mathrm{d}G_{\mathrm{m}}^{i}}{\mathrm{d}g_{\mathrm{m}}} = r_{\mathrm{m,m}}^{i} = Q_{\mathrm{m,m}}^{i} \tag{S14b}$$

Between juvenile patchmates

$$\frac{\mathrm{d}G_{\mathrm{f}}^{p}}{\mathrm{d}g_{\mathrm{f}}} = r_{\mathrm{f,f}}^{p} = Q_{\mathrm{f,f}}^{p} \tag{S15a}$$

$$\frac{\mathrm{d}G_{\mathrm{m}}^{p}}{\mathrm{d}g_{\mathrm{f}}} = r_{\mathrm{f,m}}^{p} = Q_{\mathrm{f,m}}^{p} \tag{S15b}$$

$$\frac{\mathrm{d}G_{\mathrm{f}}^{p}}{\mathrm{d}g_{\mathrm{m}}} = r_{\mathrm{m,f}}^{p} = Q_{\mathrm{f,m}}^{p} \tag{S15c}$$

$$\frac{\mathrm{d}G_{\mathrm{m}}^{p}}{\mathrm{d}g_{\mathrm{m}}} = r_{\mathrm{m,m}}^{p} = Q_{\mathrm{m,m}}^{p} \tag{S15d}$$

Between adult patchmates

For the adults on a patch, we use whole group relatedness - i.e. sampling without replacement. The relatedness between two adult females on a patch $r_{\mathrm{f,f}}^{q}$, is then equal to the probability that the same individual is sampled twice ($1/n_{\mathrm{f}}$) multiplied by their relatedness to self $r_{\mathrm{f,f}}^{i}$, and the probability two different adults are sampled ($(n_{\mathrm{f}}-1)/n_{\mathrm{f}}$), multiplied by the probability that they both did not disperse $(1-d_{\mathrm{f}})^2$, and then multiplied by the relatedness between two juvenile females $r_{\mathrm{f,f}}^{p}$. We can use this same approach to calculate the relatedness between other pairs of adults ($r_{\mathrm{f,m}}^{q}, r_{\mathrm{m,f}}^{q}, r_{\mathrm{m,m}}^{q}$).

$$\frac{\mathrm{d}G_{\mathrm{f}}^{q}}{\mathrm{d}g_{\mathrm{f}}} = r_{\mathrm{f,f}}^{q} = \left(\frac{1}{n_{\mathrm{f}}}\right) r_{\mathrm{f,f}}^{i} + \left(\frac{n_{\mathrm{f}}-1}{n_{\mathrm{f}}}\right)(1-d_{\mathrm{f}})^2 r_{\mathrm{f,f}}^{p} \tag{S16a}$$

$$\frac{\mathrm{d}G_{\mathrm{m}}^{q}}{\mathrm{d}g_{\mathrm{f}}} = r_{\mathrm{f,m}}^{q} = \phi r_{\mathrm{f,m}}^{p} \tag{S16b}$$

$$\frac{\mathrm{d}G_{\mathrm{f}}^{q}}{\mathrm{d}g_{\mathrm{m}}} = r_{\mathrm{m,f}}^{q} = \phi r_{\mathrm{m,f}}^{p} \tag{S16c}$$

$$\frac{\mathrm{d}G_{\mathrm{m}}^{q}}{\mathrm{d}g_{\mathrm{m}}} = r_{\mathrm{m,m}}^{q} = \left(\frac{1}{n_{\mathrm{m}}}\right) r_{\mathrm{m,m}}^{i} + \left(\frac{n_{\mathrm{m}}-1}{n_{\mathrm{m}}}\right)(1-d_{\mathrm{m}})^{2} r_{\mathrm{m,m}}^{p} \tag{S16d}$$

Between juveniles and the adults in their patch in the previous generation

$$\frac{\mathrm{d}G_{\mathrm{f}}^{v}}{\mathrm{d}g_{\mathrm{f}}} = r_{\mathrm{f,f}}^{v} = Q_{\mathrm{f,f}}^{v} \tag{S17a}$$

$$\frac{\mathrm{d}G_{\mathrm{m}}^{v}}{\mathrm{d}g_{\mathrm{f}}} = r_{\mathrm{f,m}}^{v} = Q_{\mathrm{f,m}}^{v} \tag{S17b}$$

$$\frac{\mathrm{d}G_{\mathrm{f}}^{v}}{\mathrm{d}g_{\mathrm{m}}} = r_{\mathrm{m,f}}^{v} = Q_{\mathrm{f,m}}^{v} \tag{S17c}$$

$$\frac{\mathrm{d}G_{\mathrm{m}}^{v}}{\mathrm{d}g_{\mathrm{m}}} = r_{\mathrm{m,m}}^{v} = Q_{\mathrm{m,m}}^{v} \tag{S17d}$$

Diploidy

Within individuals

$$\frac{\mathrm{d}G_{\mathrm{f}}^{i}}{\mathrm{d}g_{\mathrm{f}}} = r_{\mathrm{f,f}}^{i} = \sigma\left(\alpha Q_{\mathrm{f_M,f_M}}^{i} + (1-\alpha) Q_{\mathrm{f_M,f_P}}^{i}\right) + (1-\sigma)\left(\alpha Q_{\mathrm{f_M,f_P}}^{i} + (1-\alpha) Q_{\mathrm{f_P,f_P}}^{i}\right) \tag{S18a}$$

$$\frac{\mathrm{d}G_{\mathrm{m}}^{i}}{\mathrm{d}g_{\mathrm{m}}} = r_{\mathrm{m,m}}^{i} = (1-\tau)\left((1-\beta) Q_{\mathrm{m_M,m_M}}^{i} + \beta Q_{\mathrm{m_M,m_P}}^{i}\right) + \tau\left((1-\beta) Q_{\mathrm{m_M,m_P}}^{i} + \beta Q_{\mathrm{m_P,m_P}}^{i}\right) \tag{S18b}$$

Between juvenile patchmates

$$\frac{\mathrm{d}G_{\mathrm{f}}^{p}}{\mathrm{d}g_{\mathrm{f}}} = r_{\mathrm{f,f}}^{p} = \sigma\left(\alpha Q_{\mathrm{f_M,f_M}}^{p} + (1-\alpha) Q_{\mathrm{f_M,f_P}}^{p}\right) + (1-\sigma)\left(\alpha Q_{\mathrm{f_M,f_P}}^{p} + (1-\alpha) Q_{\mathrm{f_P,f_P}}^{p}\right) \tag{S19a}$$

$$\frac{\mathrm{d}G_{\mathrm{m}}^{p}}{\mathrm{d}g_{\mathrm{f}}} = r_{\mathrm{f,m}}^{p} = (1-\tau)\left(\alpha Q_{\mathrm{f_M,m_M}}^{p} + (1-\alpha) Q_{\mathrm{f_P,m_M}}^{p}\right) + \tau\left(\alpha Q_{\mathrm{f_M,m_P}}^{p} + (1-\alpha) Q_{\mathrm{f_P,m_P}}^{p}\right) \tag{S19b}$$

$$\frac{\mathrm{d}G_{\mathrm{f}}^{p}}{\mathrm{d}g_{\mathrm{m}}} = r_{\mathrm{m,f}}^{p} = \sigma\left((1-\beta) Q_{\mathrm{f_M,m_M}}^{p} + \beta Q_{\mathrm{f_M,m_P}}^{p}\right) + (1-\sigma)\left((1-\beta) Q_{\mathrm{f_P,m_M}}^{p} + \beta Q_{\mathrm{f_P,m_P}}^{p}\right) \tag{S19c}$$

$$\frac{\mathrm{d}G_{\mathrm{m}}^{p}}{\mathrm{d}g_{\mathrm{m}}} = r_{\mathrm{m,m}}^{p} = (1-\tau)\left((1-\beta) Q_{\mathrm{m_M,m_M}}^{p} + \beta Q_{\mathrm{m_M,m_P}}^{p}\right) + \tau\left((1-\beta) Q_{\mathrm{m_M,m_P}}^{p} + \beta Q_{\mathrm{m_P,m_P}}^{p}\right) \tag{S19d}$$

Between adult patchmates

$$\frac{\mathrm{d}G_{\mathrm{f}}^{q}}{\mathrm{d}g_{\mathrm{f}}} = r_{\mathrm{f,f}}^{q} = \left(\frac{1}{n_{\mathrm{f}}}\right) r_{\mathrm{f,f}}^{i} + \left(\frac{n_{\mathrm{f}}-1}{n_{\mathrm{f}}}\right)(1-d_{\mathrm{f}})^{2} r_{\mathrm{f,f}}^{p} \tag{S20a}$$

$$\frac{dG^q_{\mathrm{m}}}{dg_{\mathrm{f}}} = r^q_{\mathrm{f,m}} = \phi r^p_{\mathrm{f,m}} \tag{S20b}$$

$$\frac{dG^q_{\mathrm{f}}}{dg_{\mathrm{m}}} = r^q_{\mathrm{m,f}} = \phi r^p_{\mathrm{m,f}} \tag{S20c}$$

$$\frac{dG^q_{\mathrm{m}}}{dg_{\mathrm{m}}} = r^q_{\mathrm{m,m}} = \left(\frac{1}{n_{\mathrm{m}}}\right) r^i_{\mathrm{m,m}} + \left(\frac{n_{\mathrm{m}}-1}{n_{\mathrm{m}}}\right)(1-d_{\mathrm{m}})^2 r^p_{\mathrm{m,m}} \tag{S20d}$$

Between juveniles and the adults in their patch in the previous generation

$$\frac{dG^v_{\mathrm{f}}}{dg_{\mathrm{f}}} = r^v_{\mathrm{f,f}} = \sigma\left(\alpha Q^v_{\mathrm{f_M,f_M}} + (1-\alpha) Q^v_{\mathrm{f_M,f_P}}\right) + (1-\sigma)\left(\alpha Q^v_{\mathrm{f_M,f_P}} + (1-\alpha) Q^v_{\mathrm{f_P,f_P}}\right) \tag{S21a}$$

$$\frac{dG^v_{\mathrm{m}}}{dg_{\mathrm{f}}} = r^v_{\mathrm{f,m}} = (1-\tau)\left(\alpha Q^v_{\mathrm{f_M,m_M}} + (1-\alpha) Q^v_{\mathrm{f_P,m_M}}\right) + \tau\left(\alpha Q^v_{\mathrm{f_M,m_P}} + (1-\alpha) Q^v_{\mathrm{f_P,m_P}}\right) \tag{S21b}$$

$$\frac{dG^v_{\mathrm{f}}}{dg_{\mathrm{m}}} = r^v_{\mathrm{m,f}} = \sigma\left((1-\beta) Q^v_{\mathrm{f_M,m_M}} + \beta Q^v_{\mathrm{f_M,m_P}}\right) + (1-\sigma)\left((1-\beta) Q^v_{\mathrm{f_P,m_M}} + \beta Q^v_{\mathrm{f_P,m_P}}\right) \tag{S21c}$$

$$\frac{dG^v_{\mathrm{m}}}{dg_{\mathrm{m}}} = r^v_{\mathrm{m,m}} = (1-\tau)\left((1-\beta) Q^v_{\mathrm{m_M,m_M}} + \beta Q^v_{\mathrm{m_M,m_P}}\right) + \tau\left((1-\beta) Q^v_{\mathrm{m_M,m_P}} + \beta Q^v_{\mathrm{m_P,m_P}}\right) \tag{S21d}$$

5 Reproductive values

Reproductive value captures the asymptotic contribution that a particular class or individual makes to the ancestry of the population, thus providing a weighting of the relative importance of selection on that individual, or in that class of individuals (Fisher, 1999; Taylor, 1990; Grafen, 2006). We can compute the class reproductive values as so, let $\wp_{\mathbb{i}\leftarrow\mathbb{j}}$ be the probability that the transmitted breeding value of randomly sampled individual of class $\mathbb{i}$ came from class $\mathbb{j}$ in the previous time point. We can then write this out as a gene flow matrix T:

$$T = \begin{pmatrix} \wp_{\mathrm{f}\leftarrow\mathrm{f}} & \wp_{\mathrm{f}\leftarrow\mathrm{m}} \\ \wp_{\mathrm{m}\leftarrow\mathrm{f}} & \wp_{\mathrm{m}\leftarrow\mathrm{m}} \end{pmatrix} \tag{S22}$$

The dominant left eigenvector associated with the dominant eigenvalue of this matrix gives us the class reproductive values of males v_{m} and females v_{f}. Note that as this is a Markov matrix, the dominant eigenvalue will be 1, hence, we can solve the following equation to get our vector of class reproductive values.

$$\begin{pmatrix} v_{\mathrm{f}} & v_{\mathrm{m}} \end{pmatrix} = \begin{pmatrix} v_{\mathrm{f}} & v_{\mathrm{m}} \end{pmatrix} \begin{pmatrix} \wp_{\mathrm{f}\leftarrow\mathrm{f}} & \wp_{\mathrm{f}\leftarrow\mathrm{m}} \\ \wp_{\mathrm{m}\leftarrow\mathrm{f}} & \wp_{\mathrm{m}\leftarrow\mathrm{m}} \end{pmatrix} \tag{S23}$$

These class reproductive values provide the weights on allele frequency changes within classes, however, we may also wish to describe the relative importance of selection on the different types of transition between classes (Hamilton, 1966; Hitchcock and Gardner, 2020), i.e. on females reproduction through sons, male reproduction through daughters, or through female survival to females, etc. These weights are also referred to as elasticities in demographic analysis (de Kroon et al., 1986; Caswell, 2000; Bienvenu and Legendre, 2015). We write out the value of these different transitions by writing out the value of the class, and the probability that

a gene sampled in that class passed through a particular route in the previous generation. The reproductive value of the transition from class $\mathbb{i}$ to class $\mathbb{j}$ can be written as:

$$v_{\mathbb{i}\to\mathbb{j}} = \wp_{\mathbb{j}\leftarrow\mathbb{i}} v_{\mathbb{j}} \tag{S24}$$

Haploidy

In the case of haploidy, the probability that an individual's transmitted breeding value came from their mother in the previous generation is λ, and the probability it came from their father is $1-\lambda$. Thus our transition matrix becomes:

$$T = \begin{pmatrix} \lambda & 1-\lambda \\ \lambda & 1-\lambda \end{pmatrix} \tag{S25}$$

And thus once normalised ($\sum_{\mathbb{i}} v_{\mathbb{i}} = 1$), the reproductive values become:

$$\begin{pmatrix} v_{\mathrm{f}} & v_{\mathrm{m}} \end{pmatrix} = \begin{pmatrix} \lambda & 1-\lambda \end{pmatrix} \tag{S26}$$

And thus the reproductive values of the transitions between classes become:

$$v_{\mathrm{f}\to\mathrm{f}} = \lambda^2 \tag{S27a}$$

$$v_{\mathrm{f}\to\mathrm{m}} = \lambda(1-\lambda) \tag{S27b}$$

$$v_{\mathrm{m}\to\mathrm{f}} = (1-\lambda)\lambda \tag{S27c}$$

$$v_{\mathrm{m}\to\mathrm{m}} = (1-\lambda)^2 \tag{S27d}$$

Diploidy

Earlier, we defined $\hat{\alpha}$ to be the proportion of an individual female's transmitted breeding value that comes from her maternal-origin gene, and we defined the proportion of a individual male's transmitted breeding value that came from his paternal-origin gene to be $\hat{\beta}$. If we define the the probability that the transmitted breeding value of a female came from a female in the previous generation to be $\tilde{\alpha}$, and the probability that the transmitted breeding value of a male came from a male in the previous generation to be $\tilde{\beta}$. Then, $\tilde{\alpha} = \hat{\alpha} = \alpha$ and similarly $\tilde{\beta} = \hat{\beta} = \beta$. With this, we can write out the gene-flow matrix T as:

$$T = \begin{pmatrix} \alpha & 1-\alpha \\ 1-\beta & \beta \end{pmatrix} \tag{S28}$$

And thus the normalised class reproductive values become:

$$\begin{pmatrix} v_{\mathrm{f}} & v_{\mathrm{m}} \end{pmatrix} = \begin{pmatrix} \frac{1-\beta}{2-\alpha-\beta} & \frac{1-\alpha}{2-\alpha-\beta} \end{pmatrix} \tag{S29}$$

And the reproductive values of transitions between classes become:

$$v_{\mathrm{f}\to\mathrm{f}} = \frac{\alpha(1-\beta)}{2-\alpha-\beta} \tag{S30a}$$

$$v_{\mathrm{f}\to\mathrm{m}} = \frac{(1-\beta)(1-\alpha)}{2-\alpha-\beta} \tag{S30b}$$

$$v_{\mathrm{m}\to\mathrm{f}} = \frac{(1-\alpha)(1-\beta)}{2-\alpha-\beta} \tag{S30c}$$

$$v_{\mathrm{m}\to\mathrm{m}} = \frac{\beta(1-\alpha)}{2-\alpha-\beta} \tag{S30d}$$

6 Condition for increase

Juvenile behaviour

We first consider a locus which affects juvenile behaviour, and denote the 'transmitted' or 'genic' or 'breeding' value (Price, 1970; Falconer, 1981; Grafen, 1985) of an individual drawn at random from the population at this locus g, with g_f being the transmitted genic value of a randomly chosen female at this locus, and g_m being the transmitted genic value of a randomly chosen male. Assuming vanishing genetic variation, the condition for natural selection to favour an increase in this trait is given by:

$$\frac{\mathrm{d}W}{\mathrm{d}g} > 0 \tag{S31}$$

In a class structured population, $W = \sum_{\mathbb{i}} \sum_{\mathbb{j}} v_{\mathbb{i}\to\mathbb{j}} W_{\mathbb{i}\to\mathbb{j}}$, which is the reproductive-value weighted average of relative fitness taken across classes, with $v_{\mathbb{i}\to\mathbb{j}}$ being the reproductive value of the transition between class $\mathbb{i}$ and $\mathbb{j}$, where $\mathbb{i},\mathbb{j} \in \{\mathrm{f},\mathrm{m}\}$. Condition S31 is therefore equivalent to:

$$\sum_{\mathbb{i}} \sum_{\mathbb{j}} v_{\mathbb{i}\to\mathbb{j}} \frac{\mathrm{d}W_{\mathbb{i}\to\mathbb{j}}}{\mathrm{d}g_{\mathbb{i}}} > 0 \tag{S32}$$

In our case, we have two classes (f and m), and so can write out as:

$$v_{\mathrm{f}\to\mathrm{m}} \frac{\mathrm{d}W_{\mathrm{f}\to\mathrm{m}}}{\mathrm{d}g_\mathrm{f}} + v_{\mathrm{f}\to\mathrm{f}} \frac{\mathrm{d}W_{\mathrm{f}\to\mathrm{f}}}{\mathrm{d}g_\mathrm{f}} + v_{\mathrm{m}\to\mathrm{f}} \frac{\mathrm{d}W_{\mathrm{m}\to\mathrm{f}}}{\mathrm{d}g_\mathrm{m}} + v_{\mathrm{m}\to\mathrm{m}} \frac{\mathrm{d}W_{\mathrm{m}\to\mathrm{m}}}{\mathrm{d}g_\mathrm{m}} > 0 \tag{S33}$$

Using the chain rule, we can then expand this out to:

$$\begin{aligned}
&v_{\mathrm{f}\to\mathrm{f}} \left(\frac{\partial W_{\mathrm{f}\to\mathrm{f}}}{\partial x} \frac{\mathrm{d}x}{\mathrm{d}g_\mathrm{f}} + \frac{\partial W_{\mathrm{f}\to\mathrm{f}}}{\partial y_\mathrm{f}} \frac{\mathrm{d}y_\mathrm{f}}{\mathrm{d}g_\mathrm{f}} + \frac{\partial W_{\mathrm{f}\to\mathrm{f}}}{\partial y_\mathrm{m}} \frac{\mathrm{d}y_\mathrm{m}}{\mathrm{d}g_\mathrm{f}} \right) + \\
&v_{\mathrm{f}\to\mathrm{m}} \left(\frac{\partial W_{\mathrm{f}\to\mathrm{m}}}{\partial x} \frac{\mathrm{d}x}{\mathrm{d}g_\mathrm{f}} + \frac{\partial W_{\mathrm{f}\to\mathrm{m}}}{\partial y_\mathrm{f}} \frac{\mathrm{d}y_\mathrm{f}}{\mathrm{d}g_\mathrm{f}} + \frac{\partial W_{\mathrm{f}\to\mathrm{m}}}{\partial y_\mathrm{m}} \frac{\mathrm{d}y_\mathrm{m}}{\mathrm{d}g_\mathrm{f}} \right) + \\
&v_{\mathrm{m}\to\mathrm{f}} \left(\frac{\partial W_{\mathrm{m}\to\mathrm{f}}}{\partial x} \frac{\mathrm{d}x}{\mathrm{d}g_\mathrm{m}} + \frac{\partial W_{\mathrm{m}\to\mathrm{f}}}{\partial y_\mathrm{f}} \frac{\mathrm{d}y_\mathrm{f}}{\mathrm{d}g_\mathrm{m}} + \frac{\partial W_{\mathrm{m}\to\mathrm{f}}}{\partial y_\mathrm{m}} \frac{\mathrm{d}y_\mathrm{m}}{\mathrm{d}g_\mathrm{m}} \right) + \\
&v_{\mathrm{m}\to\mathrm{m}} \left(\frac{\partial W_{\mathrm{m}\to\mathrm{m}}}{\partial x} \frac{\mathrm{d}x}{\mathrm{d}g_\mathrm{m}} + \frac{\partial W_{\mathrm{m}\to\mathrm{m}}}{\partial y_\mathrm{f}} \frac{\mathrm{d}y_\mathrm{f}}{\mathrm{d}g_\mathrm{m}} + \frac{\partial W_{\mathrm{m}\to\mathrm{m}}}{\partial y_\mathrm{m}} \frac{\mathrm{d}y_\mathrm{m}}{\mathrm{d}g_\mathrm{m}} \right) > 0
\end{aligned} \tag{S34}$$

Substituting in our marginal fitness effects, and rewriting the derivatives as relatedness coefficients, our condition simplifies down to:

$$\begin{aligned}
&v_\mathrm{f} \left(-c_{\mathrm{fj}} (r^i_{\mathrm{ff}} - k_\mathrm{f} r^p_{\mathrm{ff}}) + (1-k_\mathrm{f})(b_{\mathrm{fj}} r^p_{\mathrm{ff}} + b_{\mathrm{mj}} r^p_{\mathrm{fm}}) \right) + \\
&v_\mathrm{m} \left(-c_{\mathrm{mj}} (r^i_{\mathrm{mm}} - k_\mathrm{m} r^p_{\mathrm{mm}}) + (1-k_\mathrm{m})(b_{\mathrm{fj}} r^p_{\mathrm{mf}} + b_{\mathrm{mj}} r^p_{\mathrm{mm}}) \right) > 0
\end{aligned} \tag{S35}$$

Where $k_{\rm f}$ is the scale of competition for females, and $k_{\rm m}$ is the scale of competition for males, i.e. the degree to which competition is occurring locally (Frank, 1998). We can then make some simplifications to generate the potentials for altruism seen in the main text.

Female specific behaviour

We first consider female-specific behaviour, in which case females may have marginal fitness effects upon self and others (i.e. $c_{\rm fj} = c; b_{\rm fj} = b$), but males have no fitness effects (i.e. $b_{\rm mj}, c_{\rm mj} = 0$). In which case the above equation simplifies down to:

$$-c[v_{\rm f}(r^i_{\rm ff} - k_{\rm f} r^p_{\rm ff})] + b[(1-k_{\rm f})\, v_{\rm f} r^p_{\rm ff} + (1-k_{\rm m})\, v_{\rm m} r^p_{\rm mf}] > 0 \tag{S36}$$

We can then rearrange this condition into a dimensionless potential for altruism (Gardner, 2010), where $c/b < A$. Note that this is similar to the κ of Lehmann and Rousset (2010). In this case:

$$A = \frac{(1-k_{\rm f})\, v_{\rm f} r^p_{\rm ff} + (1-k_{\rm m})\, v_{\rm m} r^p_{\rm mf}}{v_{\rm f}(r^i_{\rm ff} - k_{\rm f} r^p_{\rm ff})} \tag{S37}$$

We can then plug in the specific values for the relatedness coefficients, reproductive values, and scales of competition generated from our different inheritance systems and assumptions about demography. Under sex-symmetric dispersal ($d_{\rm f} = d_{\rm m} = d$, $\kappa_{\rm f} = \kappa_{\rm m} = (1-d)^2$) and an even sex ratio of adults breeders on each patch ($n_{\rm f} = n_{\rm m} = n$), then for diploidy, arrhenotoky, male PGE, and paterothylotoky, the potential for altruism simplifies down to:

$$A = \frac{1}{n} \tag{S38}$$

Which recovers the result found by Gardner (2010) in his analysis of juvenile altruism. For female maternal genome elimination:

$$A = \frac{4-(1-d)^2}{\left(4-(1-d)^2\right) n(1-\sigma) + (1-d)^2\sigma} \tag{S39}$$

Where σ represents the proportion of expression that comes from the maternal-origin gene copy in females. With this parameter we may then manipulate the degree of "control" that the maternal-origin versus paternal-origin copies have over the female phenotype. For example, if the maternal-origin gene copy exclusively determines the phenotype then $\sigma = 1$. Alternatively, if - in females - the paternal-origin copy exclusively exerts "control" over the phenotype then $\sigma = 0$.

For haploidy:

$$A = \frac{(1-\lambda)^2 + \lambda^2}{n\lambda} \tag{S40}$$

Where again, λ represents the probability that an offspring inherits their genome from their mother rather than father. When $\lambda = 1/2$, then once again $A = 1/n$.

Male specific behaviour

Similarly, for a behaviour that is male specific (i.e. $c_{\rm fj}, b_{\rm fj} = 0; b_{\rm mj} = b; c_{\rm mj} = c$):

$$A = \frac{(1-k_{\rm f})\, v_{\rm f} r^p_{\rm fm} + (1-k_{\rm m})\, v_{\rm m} r^p_{\rm mm}}{v_{\rm m}(r^i_{\rm mm} - k_{\rm m} r^p_{\rm mm})} \tag{S41}$$

We can then plug in the specific values for the relatedness coefficients and reproductive values generated from our different inheritance systems. Under sex-symmetric dispersal and an even sex ratio of adults on each patch

($d_\mathrm{f} = d_\mathrm{m} = d$ and $n_\mathrm{f} = n_\mathrm{m} = n$), then for diploidy, arrhenotoky, paterothylotoky, and female maternal-genome elimination:

$$A = \frac{1}{n} \tag{S42}$$

However, under male PGE, the potential for juvenile altruism is:

$$A = \frac{4-(1-d)^2}{\left(4-(1-d)^2\right)n(1-\tau)+(1-d)^2\tau} \tag{S43}$$

Which when $\tau = 1/2$ -i.e. when maternal-origin genes and paternal-origin genes contribute equally to the phenotype in males - recovers equation 1 of the main text. For haploidy, the potential for altruism is given by:

$$A = \frac{(1-\lambda)^2+\lambda^2}{n(1-\lambda)} \tag{S44}$$

Where again, λ represents the probability that an offspring inherits their genome from their mother rather than father.

Both sexes express the behaviour

For a behaviour expressed by both sexes (i.e. $c_\mathrm{fj} = c_\mathrm{mj} = c; b_\mathrm{fj} = b_\mathrm{mj} = b$):

$$A = \frac{(1-k_\mathrm{f})v_\mathrm{f}\left(r^p_\mathrm{ff}+r^p_\mathrm{fm}\right)+(1-k_\mathrm{m})v_\mathrm{m}\left(r^p_\mathrm{mf}+r^p_\mathrm{mm}\right)}{v_\mathrm{f}\left(r^i_\mathrm{ff}-k_\mathrm{f}r^p_\mathrm{ff}\right)+v_\mathrm{m}\left(r^i_\mathrm{mm}-k_\mathrm{m}r^p_\mathrm{mm}\right)} \tag{S45}$$

Once again, plugging in the values for relatedness and reproductive value under sex-neutral demography ($d_\mathrm{f} = d_\mathrm{m} = d$ and $n_\mathrm{f} = n_\mathrm{m} = n$), for diploidy, arrhenotoky, and paterothylotoky:

$$A = \frac{1}{n} \tag{S46}$$

Note that this recovers the results of Gardner (2010). Whilst there is an apparent factor of two difference between the these results this only arises because of a slight difference in how the b's are defined, otherwise they are equivalent. For male PGE:

$$A = \frac{8-2(1-d)^2}{\left(4-(1-d)^2\right)n(2-\tau)+(1-d)^2\tau} \tag{S47}$$

For female MGE:

$$A = \frac{8-2(1-d)^2}{\left(4-(1-d)^2\right)n(2-\sigma)+(1-d)^2\sigma} \tag{S48}$$

And for haploidy:

$$A = 2\frac{(1-\lambda)^2+\lambda^2}{n} \tag{S49}$$

Full expressions for arbitrary values of d_f, d_m,n_f and n_m are unwieldy. However, we plot results for the some intermediate scenarios in Figures S2-S3 and S6-S7.

Adult behaviour

We follow a similar procedure for adult specific behaviour. In which case we find the condition for a trait to increase is given by:

$$\begin{aligned} &v_\mathrm{f}\left(-c_\mathrm{fa}(r^i_\mathrm{ff}-k_\mathrm{f}r^v_\mathrm{ff})+b_\mathrm{fa}(r^P_\mathrm{ff}-k_\mathrm{f}r^v_\mathrm{ff})+\frac{1}{\gamma}(b_\mathrm{ma}-c_\mathrm{ma})(r^P_\mathrm{fm}-k_\mathrm{f}r^v_\mathrm{fm})\right)+ \\ &v_\mathrm{m}\left(-c_\mathrm{ma}(r^i_\mathrm{mm}-k_\mathrm{m}r^v_\mathrm{mm})+b_\mathrm{ma}(r^P_\mathrm{mm}-k_\mathrm{m}r^v_\mathrm{mm})+\gamma(b_\mathrm{fa}-c_\mathrm{fa})(r^P_\mathrm{mf}-k_\mathrm{m}r^v_\mathrm{mf})\right)>0\end{aligned} \tag{S50}$$

Where $\gamma = n_{\mathrm{f}}/n_{\mathrm{m}}$. As before, we can rewrite this as a potential for altruism.

Female specific behaviour

First, for behaviour expressed solely by females (i.e. $c_{\mathrm{fa}} = c; b_{\mathrm{fa}} = b; b_{\mathrm{ma}}, c_{\mathrm{ma}} = 0$), the potential for altruism becomes:

$$A = \frac{v_{\mathrm{f}}(r^P_{\mathrm{ff}} - k_{\mathrm{f}} r^v_{\mathrm{ff}}) + v_{\mathrm{m}}\gamma(r^P_{\mathrm{mf}} - k_{\mathrm{m}} r^v_{\mathrm{mf}})}{v_{\mathrm{f}}(r^i_{\mathrm{ff}} - k_{\mathrm{f}} r^v_{\mathrm{ff}}) + v_{\mathrm{m}}\gamma(r^P_{\mathrm{mf}} - k_{\mathrm{m}} r^v_{\mathrm{mf}})} \tag{S51}$$

Which, when we plug in our values for the relatedness coefficients and reproductive values, then under sex-neutral demography ($d_{\mathrm{f}} = d_{\mathrm{m}} = d$ and $n_{\mathrm{f}} = n_{\mathrm{m}} = n$), the results for diploidy, arrhenotoky, paterothylotoky, and male PGE become:

$$A = \frac{1}{n} \tag{S52}$$

For female MGE:

$$A = \frac{\left(1-(1-d)^2\right)\sigma\left(4n-(n+1)(1-d)^2\right) - n\left(4-(1-d)^2\right)}{\left(n-(1-d)^2\right)\sigma\left(4n-(n+1)(1-d)^2\right) - n^2\left(4-(1-d)^2\right)} \tag{S53}$$

And for haploidy:

$$A = \frac{\lambda-(1-d)^2(1-2(1-\lambda))(1-\lambda)}{\lambda n-(1-d)^2(1-2(1-\lambda))(1-\lambda)} \tag{S54}$$

Male specific behaviour

For male specific behaviour (i.e. $c_{\mathrm{fa}}, b_{\mathrm{fa}} = 0; b_{\mathrm{ma}} = b; c_{\mathrm{ma}} = c$), the potential for altruism is:

$$A = \frac{v_{\mathrm{m}}(r^P_{\mathrm{mm}} - k_{\mathrm{m}} r^v_{\mathrm{mm}}) + v_{\mathrm{f}}(1/\gamma)(r^P_{\mathrm{fm}} - k_{\mathrm{f}} r^v_{\mathrm{fm}})}{v_{\mathrm{m}}(r^i_{\mathrm{mm}} - k_{\mathrm{m}} r^v_{\mathrm{mm}}) + v_{\mathrm{f}}(1/\gamma)(r^P_{\mathrm{fm}} - k_{\mathrm{f}} r^v_{\mathrm{fm}})} \tag{S55}$$

Which again, when we substitute in the appropriate relatedness coefficients and reproductive values, and assume sex-neutral demography ($d_{\mathrm{f}} = d_{\mathrm{m}} = d$ and $n_{\mathrm{f}} = n_{\mathrm{m}} = n$), simplify down to:

$$A = \frac{1}{n} \tag{S56}$$

For diploidy, arrhenotoky, paterothylotoky, and female MGE. Whilst for male PGE:

$$A = \frac{\left(1-(1-d)^2\right)\tau\left(4n-(n+1)(1-d)^2\right) - n\left(4-(1-d)^2\right)}{\left(n-(1-d)^2\right)\tau\left(4n-(n+1)(1-d)^2\right) - n^2\left(4-(1-d)^2\right)} \tag{S57}$$

And for haploidy:

$$A = \frac{(1-\lambda)-(1-d)^2(1-2\lambda)\lambda}{(1-\lambda)n-(1-d)^2(1-2\lambda)\lambda} \tag{S58}$$

Both sexes express the behaviour

And for behaviour expressed by both sexes (i.e. $c_{\mathrm{fa}} = c_{\mathrm{ma}} = c; b_{\mathrm{fa}} = b_{\mathrm{ma}} = b$):

$$A = \frac{v_{\mathrm{f}}\left((r^P_{\mathrm{ff}} - k_{\mathrm{f}} r^v_{\mathrm{ff}}) + (1/\gamma)(r^P_{\mathrm{fm}} - k_{\mathrm{f}} r^v_{\mathrm{fm}})\right) + v_{\mathrm{m}}\left((r^P_{\mathrm{mm}} - k_{\mathrm{m}} r^v_{\mathrm{mm}}) + \gamma(r^P_{\mathrm{mf}} - k_{\mathrm{m}} r^v_{\mathrm{mf}})\right)}{v_{\mathrm{f}}\left((r^i_{\mathrm{ff}} - k_{\mathrm{f}} r^v_{\mathrm{ff}}) + (1/\gamma)(r^P_{\mathrm{fm}} - k_{\mathrm{f}} r^v_{\mathrm{fm}})\right) + v_{\mathrm{m}}\left((r^i_{\mathrm{mm}} - k_{\mathrm{m}} r^v_{\mathrm{mm}}) + \gamma(r^P_{\mathrm{mf}} - k_{\mathrm{m}} r^v_{\mathrm{mf}})\right)} \tag{S59}$$

Once again, putting in the specific values for the relatedness coefficients and reproductive values, we find that under sex-symmetric dispersal and with an even sex-ratio of breeders then for diploidy, arrhenotoky, and paterothylotoky:

$$A = \frac{1}{n} \tag{S60}$$

For female MGE:

$$A = \frac{\left(4-(1-d)^2\right) n \left(2-\left(1-(1-d)^2\right)\sigma\right) + \left(1-(1-d)^2\right)(1-d)^2\sigma}{\left(4-(1-d)^2\right) n^2(2-\sigma) + \left(5-(1-d)^2\right)(1-d)^2 n\sigma - (1-d)^4\sigma} \tag{S61}$$

For male PGE:

$$A = \frac{\left(4-(1-d)^2\right) n \left(2-\left(1-(1-d)^2\right)\tau\right) + \left(1-(1-d)^2\right)(1-d)^2\tau}{\left(4-(1-d)^2\right) n^2(2-\tau) + \left(5-(1-d)^2\right)(1-d)^2 n\tau - (1-d)^4\tau} \tag{S62}$$

And for haploidy:

$$A = \frac{1+(1-d)^2(1-2\lambda)^2}{n+(1-d)^2(1-2\lambda)^2} \tag{S63}$$

Once again, full expressions for the sex-biased scenarios are cumbersome to present, but instead we plot some of these scenarios in Figures S4-S5 and S8-S9.

7 Figures

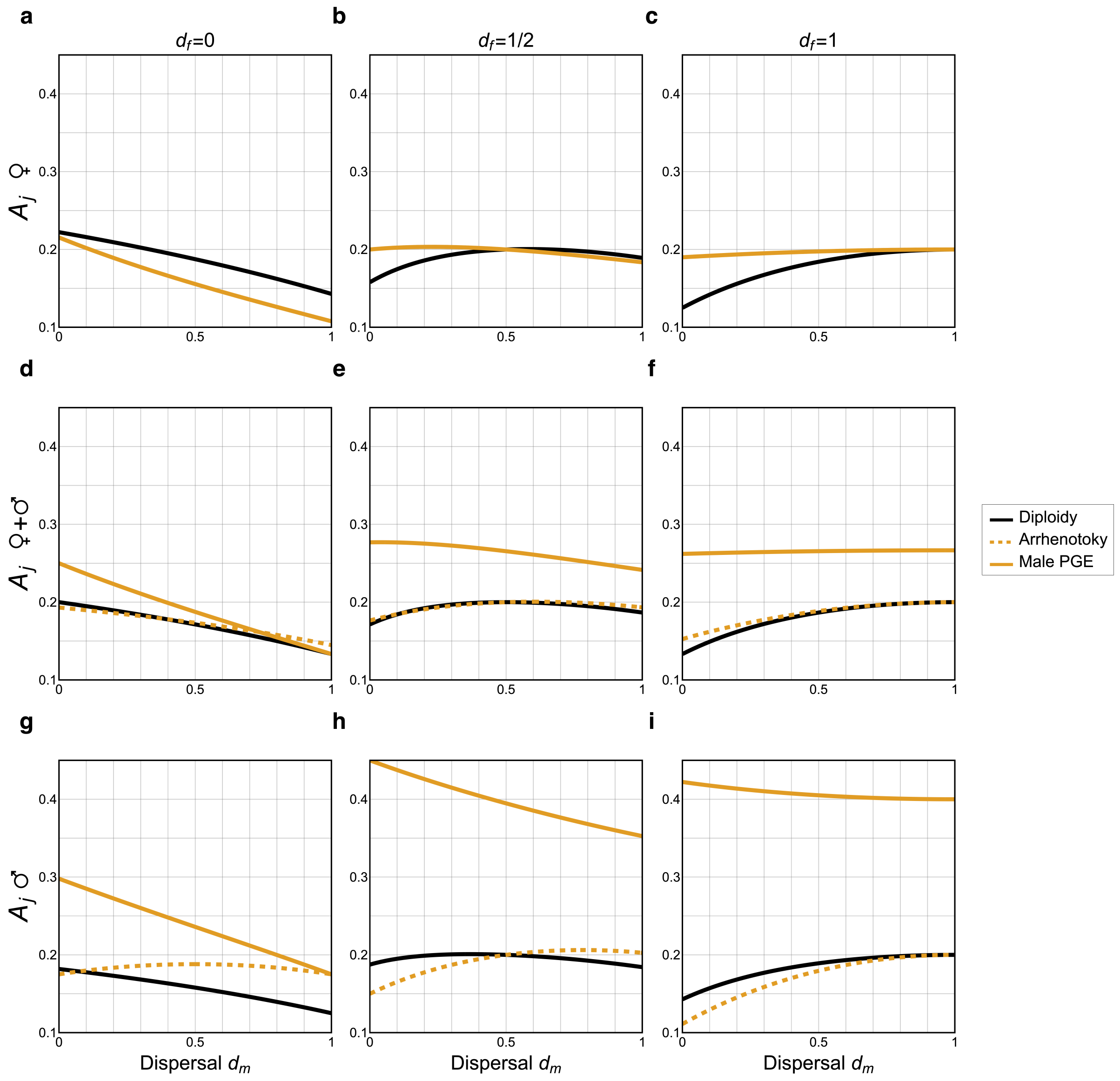

Figure S2: **The potential for altruism amongst juveniles A_j when there are sex-biases in dispersal**. In all panels $n_\mathrm{f} = n_\mathrm{m} = 5$ and $d_\mathrm{m} = 1/2$. For the case of male PGE we assume $\tau = 1/2$. Methods to regenerate these plots can be found in SM§1-6.

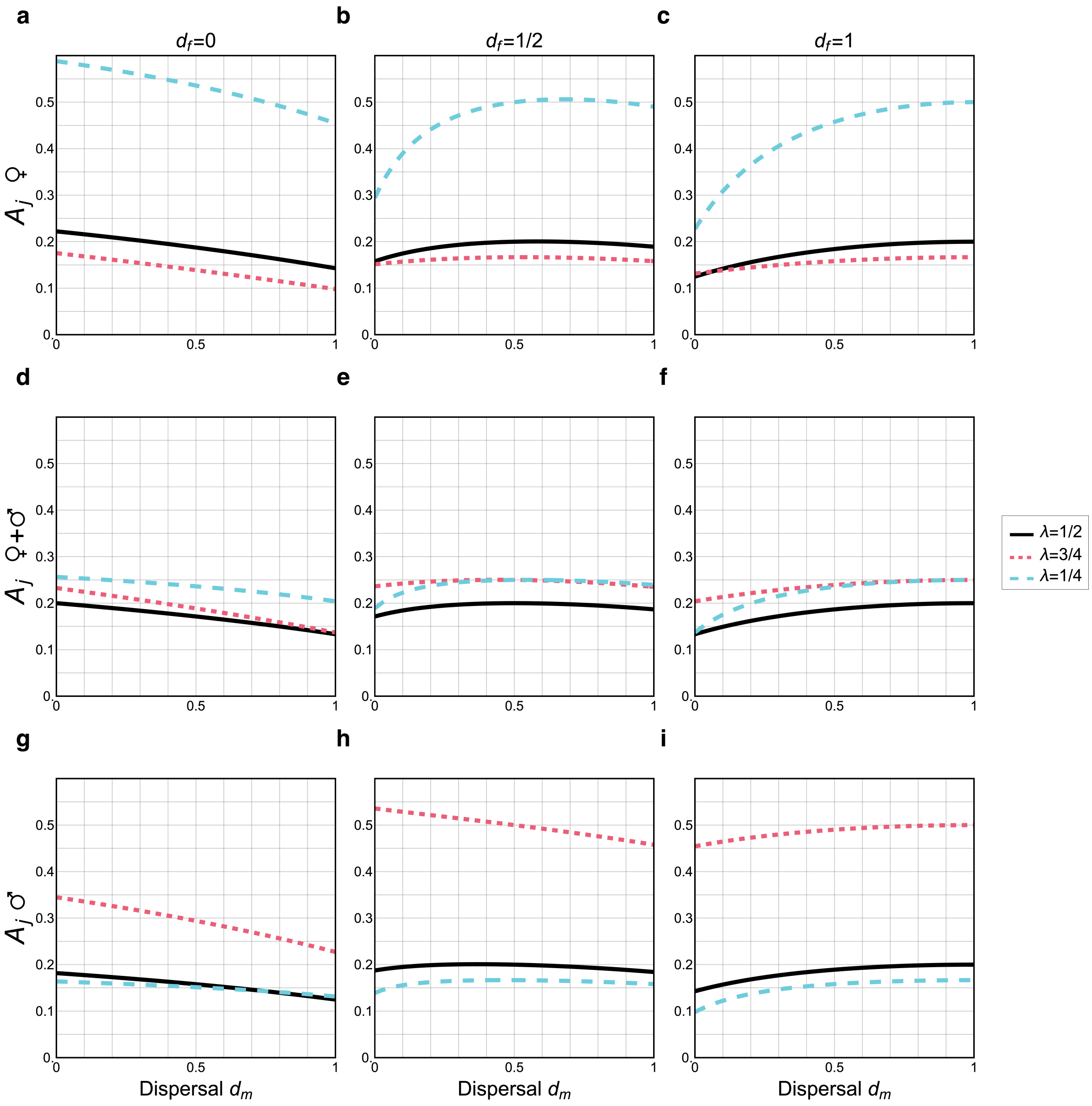

Figure S3: **The potential for altruism amongst haploid juveniles A_{j} when there are sex-biases in dispersal, and varying extents of sex-biased transmission λ.** In all panels $n_{\mathrm{f}} = n_{\mathrm{m}} = 5$ and $d_{\mathrm{m}} = 1/2$. Methods to regenerate these plots can be found in SM§1-6.

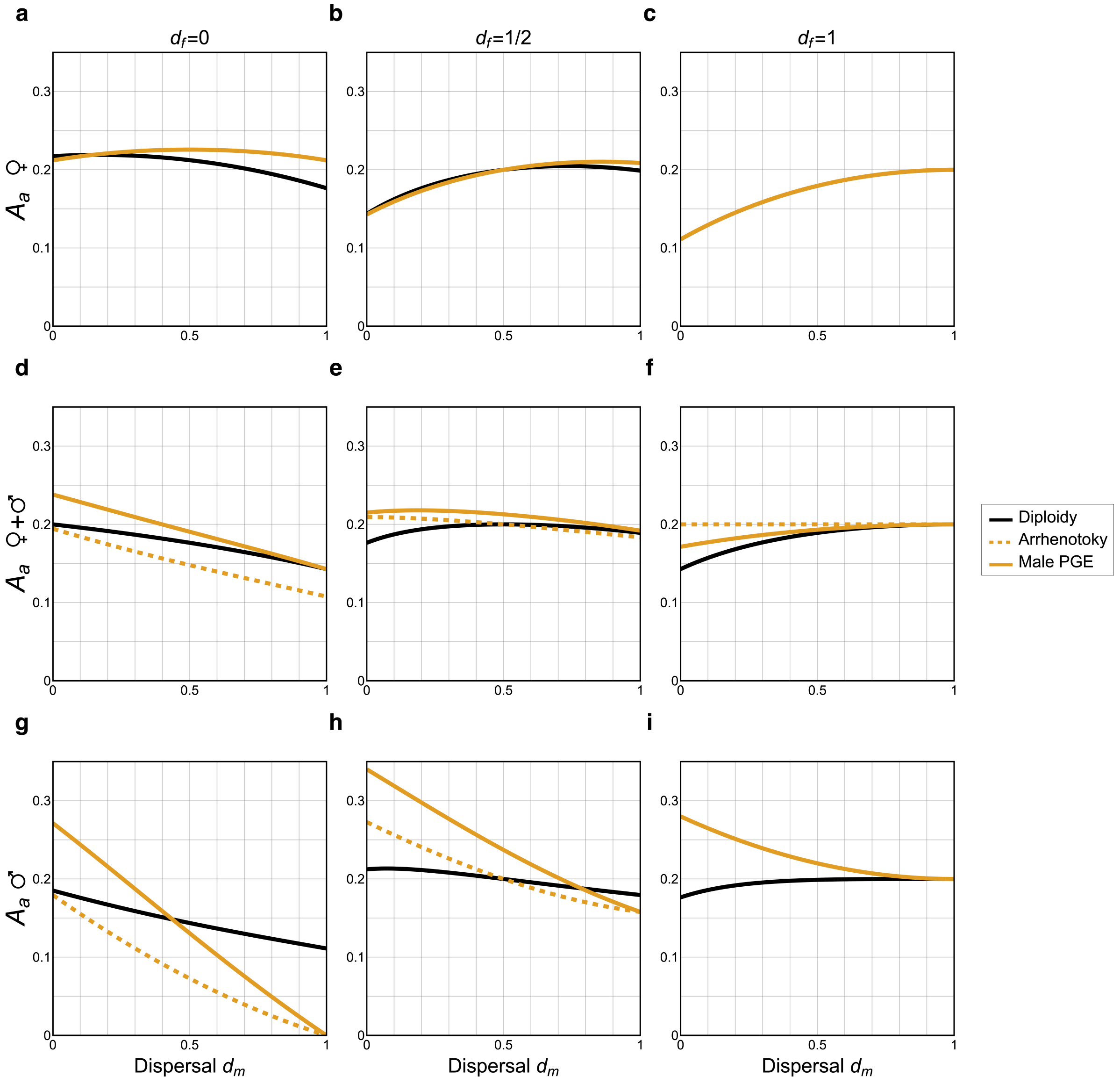

Figure S4: **The potential for altruism amongst adults A_a when there are sex-biases in dispersal**. In all panels $n_\text{f} = n_\text{m} = 5$ and $d_\text{m} = 1/2$. For the case of male PGE we assume $\tau = 1/2$. Methods to regenerate these plots can be found in SM§1-6.

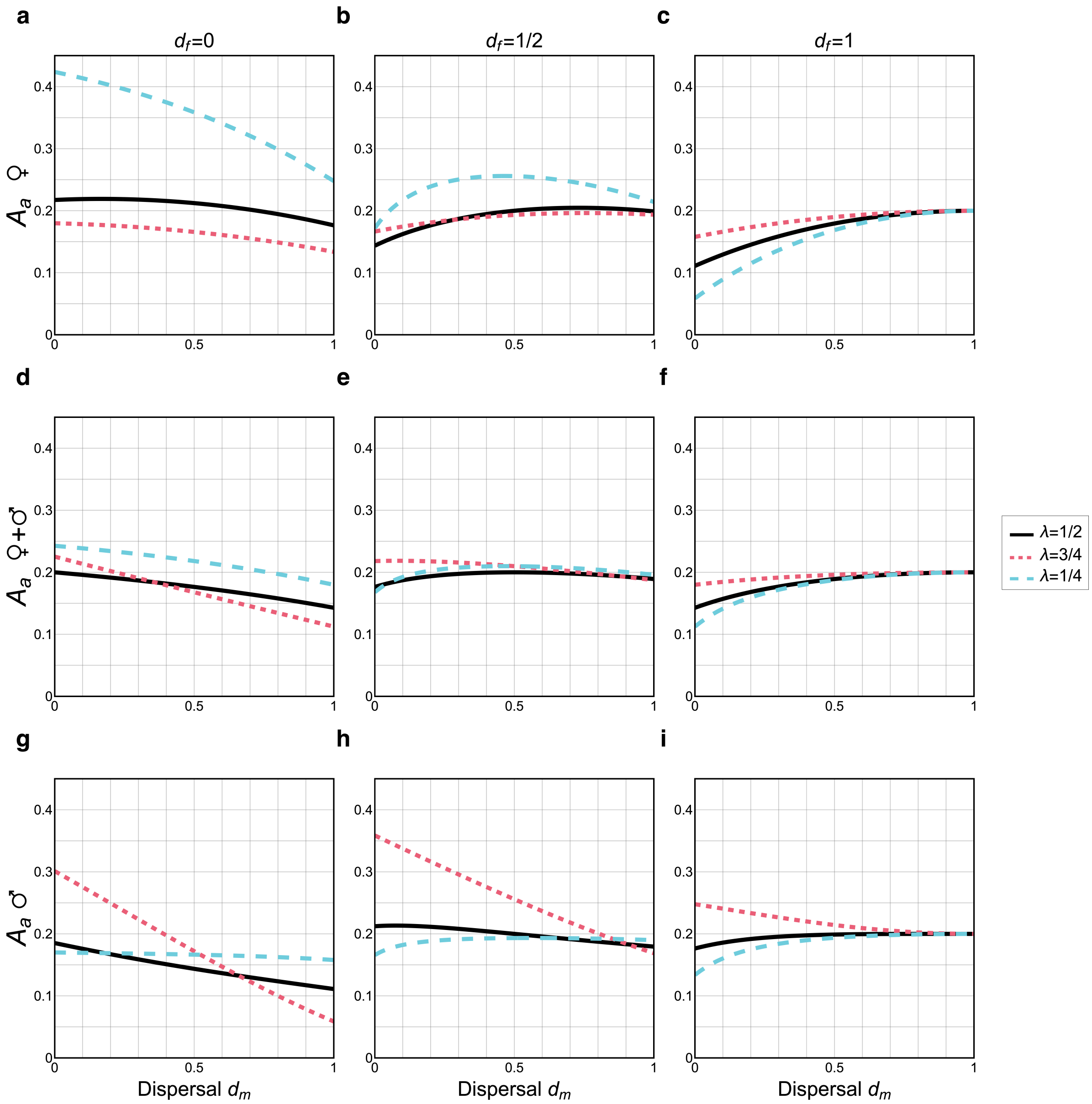

Figure S5: **The potential for altruism amongst haploid adults A_a when there are sex-biases in dispersal, and varying extents of sex-biased transmission λ.** In all panels $n_f = n_m = 5$ and $d_m = 1/2$. Methods to regenerate these plots can be found in SM§1-6.

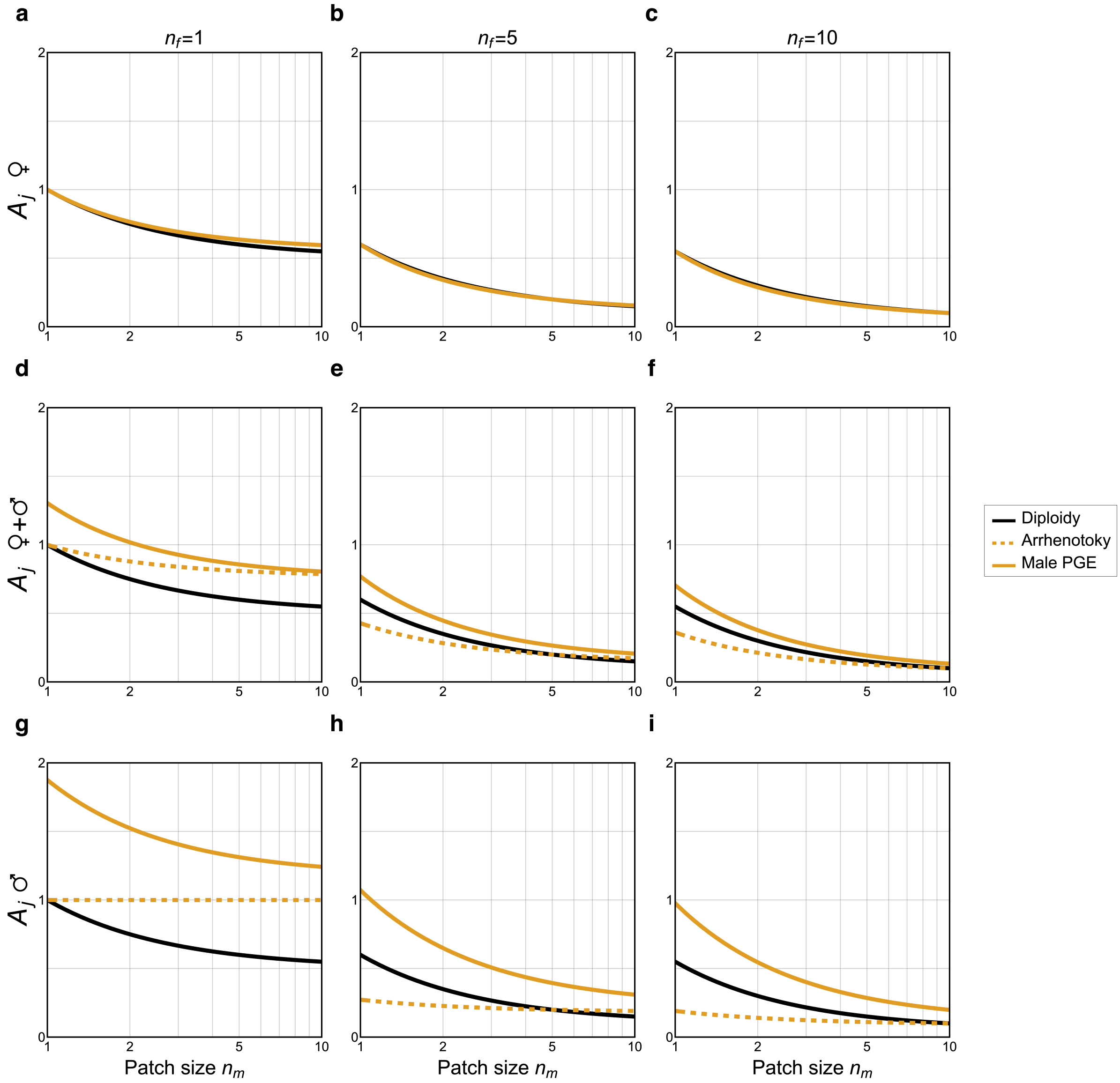

Figure S6: **The potential for altruism amongst juveniles A_j when there are sex-biases in the number of adult breeders per patch**. In all panels $d_f = d_m = 1/2$ and $n_m = 5$. For the case of male PGE we assume $\tau = 1/2$. Methods to regenerate these plots can be found in SM§1-6.

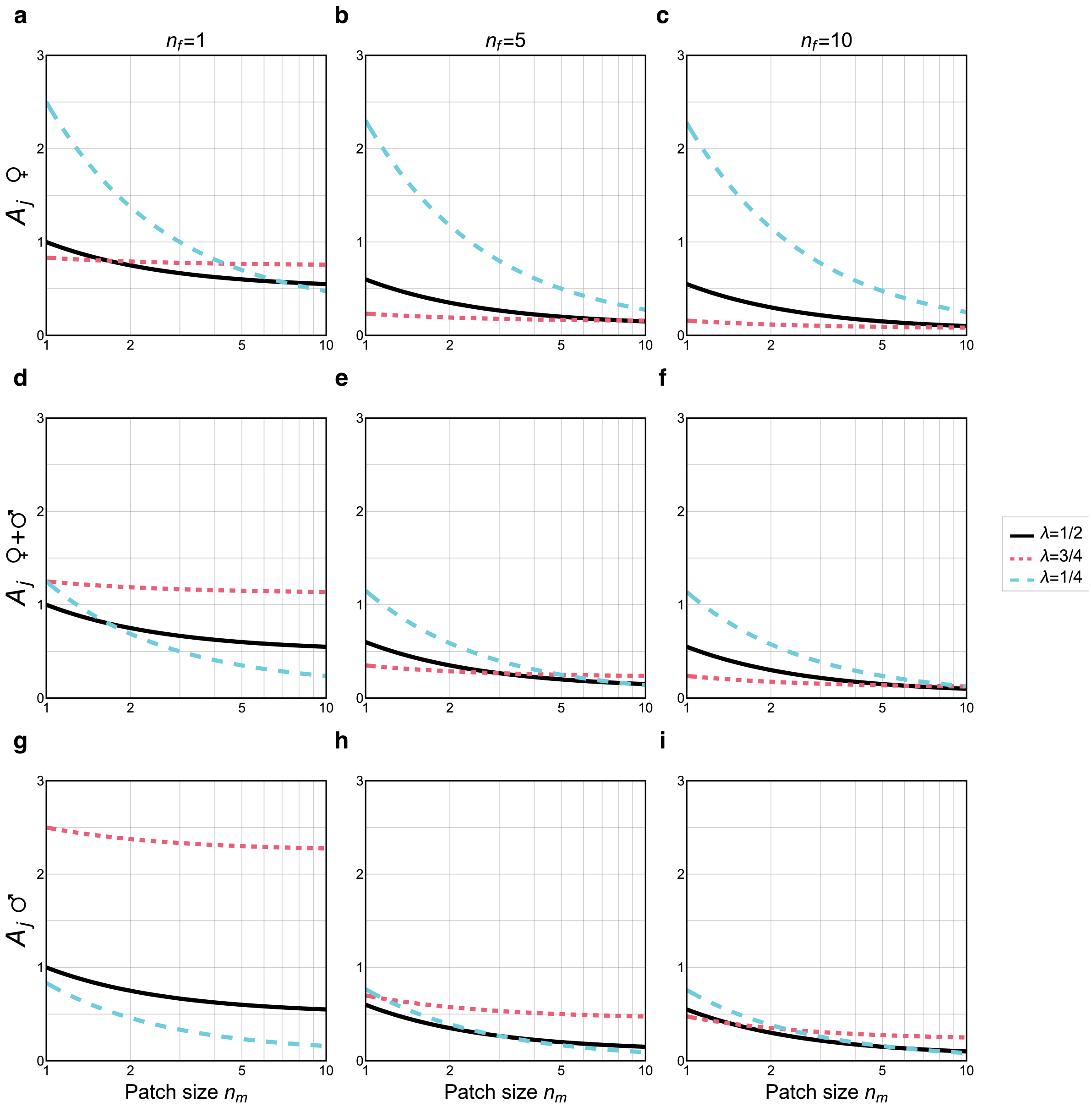

Figure S7: **The potential for altruism amongst haploid juveniles A_j when there are sex-biases in the number of adult breeders per patch, and varying extents of sex-biased transmission λ.** In all panels $d_f = d_m = 1/2$ and $n_m = 5$. Methods to regenerate these plots can be found in SM§1-6.

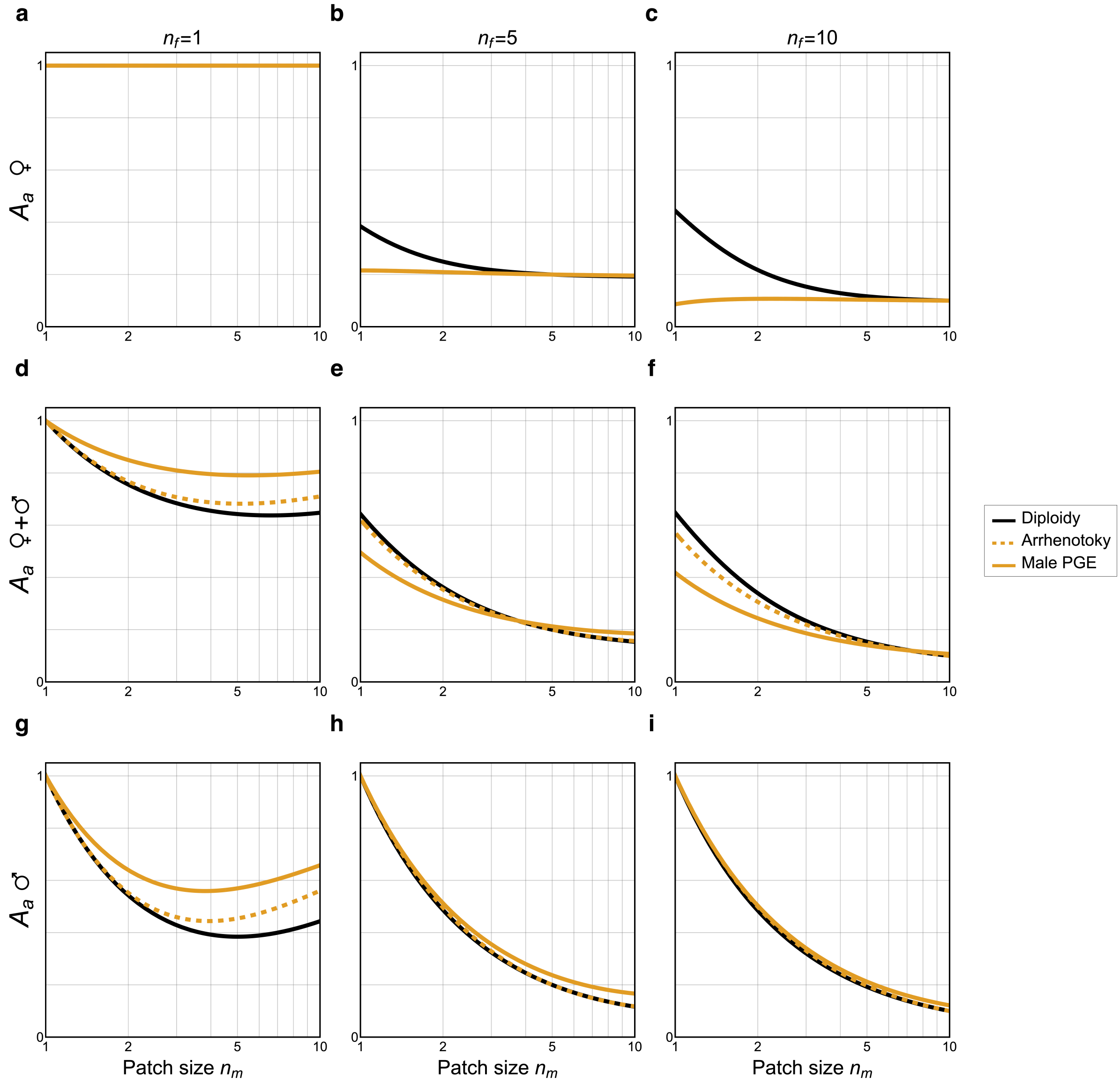

Figure S8: **The potential for altruism amongst adults A_a when there are sex-biases in the number of adult breeders per patch**. In all panels $d_f = d_m = 1/2$ and $n_m = 5$. For the case of male PGE we assume $\tau = 1/2$. Methods to regenerate these plots can be found in SM§1-6.

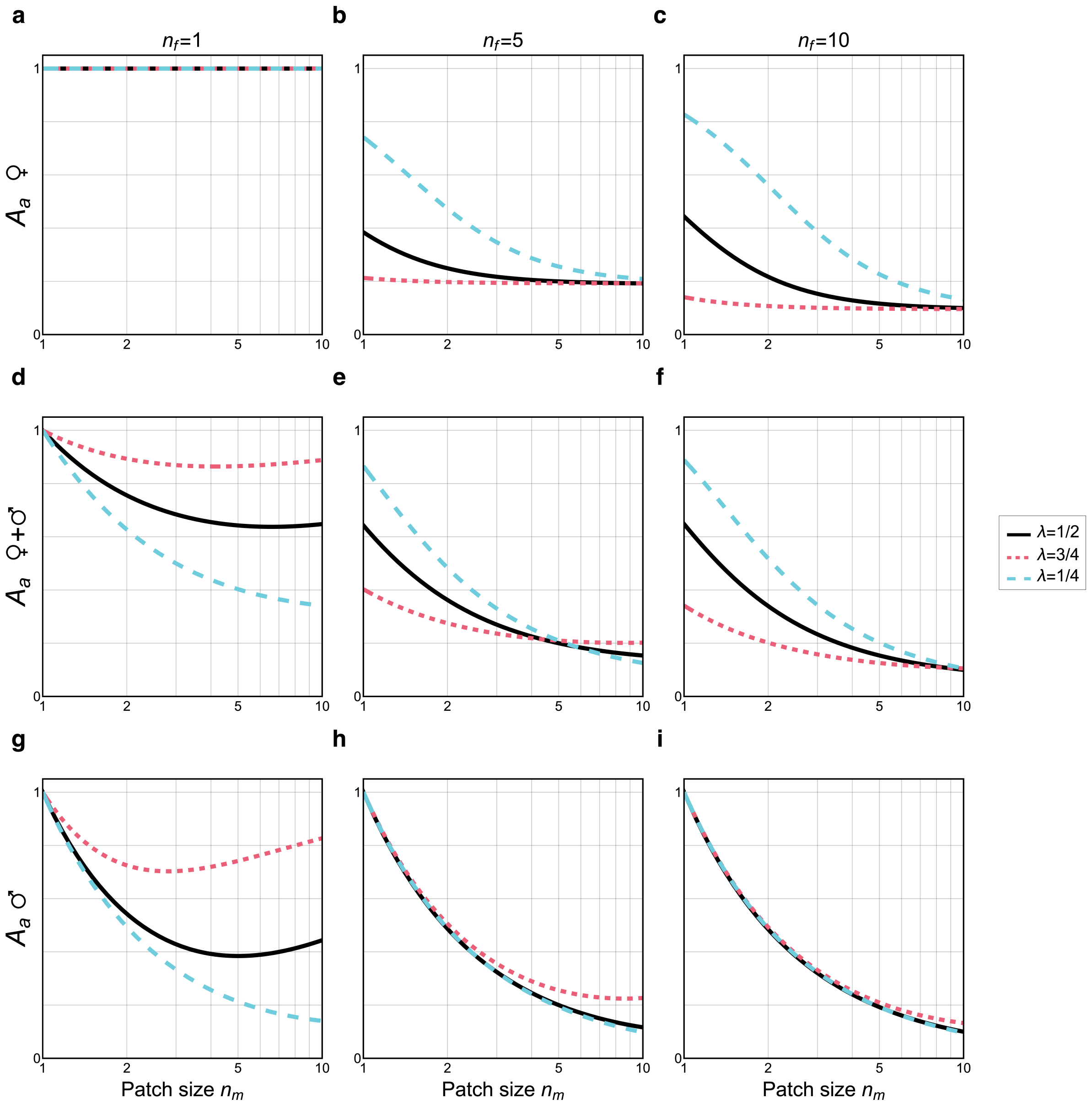

Figure S9: **The potential for altruism amongst haploid adults A_a when there are sex-biases in the number of adult breeders per patch, and varying extents of sex-biased transmission λ.** In all panels $d_\mathrm{f} = d_\mathrm{m} = 1/2$ and $n_\mathrm{m} = 5$. Methods to regenerate these plots can be found in SM§1-6.

References

Bienvenu, F. and S. Legendre (2015). “A new approach to the generation time in matrix population models”. In: *The American Naturalist* 185.6, pp. 834–843.

Caswell, H. (2000). *Matrix population models*. Vol. 1. Sinauer Sunderland, MA, USA.

De Kroon, H., A. Plaisier, J. van Groenendael, and H. Caswell (1986). “Elasticity: The Relative Contribution of Demographic Parameters to Population Growth Rate”. In: *Ecology* 67.5, pp. 1427–1431.

Falconer, D. (1981). *Introduction to quantitative genetics. 2nd ed.* Longman.

Fisher, R. (1999). *The genetical theory of natural selection: a complete variorum edition*. Oxford University Press.

Frank, S. A. (1998). *Foundations of social evolution*. Princeton University Press.

Gardner, A. (2010). “Sex-biased dispersal of adults mediates the evolution of altruism among juveniles”. In: *Journal of Theoretical Biology* 262.2, pp. 339–345.

Gardner, A., S. A. West, and G. Wild (2011). “The genetical theory of kin selection”. In: *Journal of evolutionary biology* 24.5, pp. 1020–1043.

Grafen, A. (1985). “A geometric view of relatedness”. In: *Oxford surveys in evolutionary biology*. Vol. 2. Chap. 2, pp. 28–89.

Grafen, A. (2006). “A theory of Fisher’s reproductive value”. In: *Journal of mathematical biology* 53.1, pp. 15–60.

Hamilton, W. D. (1966). “The moulding of senescence by natural selection”. In: *Journal of theoretical biology* 12.1, pp. 12–45.

Hitchcock, T. J. and A. Gardner (2020). “A gene’s-eye view of sexual antagonism”. In: *Proceedings of the Royal Society B* 287.1932, p. 20201633.

Johnstone, R. A. and M. A. Cant (2008). “Sex differences in dispersal and the evolution of helping and harming”. In: *The American Naturalist* 172.3, pp. 318–330.

Lehmann, L. and F. Rousset (2010). “How life history and demography promote or inhibit the evolution of helping behaviours”. In: *Philosophical transactions of the Royal Society B: biological sciences* 365.1553, pp. 2599–2617.

Price, G. R. (1970). “Selection and covariance.” In: *Nature* 227, pp. 520–521.

Rousset, F. (2004). *Genetic Structure and Selection in Subdivided Populations*. Vol. 40. Princeton University Press.

Taylor, P. D. (1990). “Allele-frequency change in a class-structured population”. In: *The American Naturalist* 135.1, pp. 95–106.
